# Integrative metabolic and transcriptomic profiling of prostate cancer tissue containing reactive stroma

**DOI:** 10.1038/s41598-018-32549-1

**Published:** 2018-09-24

**Authors:** Maria K. Andersen, Kjersti Rise, Guro F. Giskeødegård, Elin Richardsen, Helena Bertilsson, Øystein Størkersen, Tone F. Bathen, Morten Rye, May-Britt Tessem

**Affiliations:** 1 Department of Circulation and Medical Imaging, NTNU - Norwegian University of Science and Technology, Trondheim, Norway; 2 Department of Clinical and Molecular Medicine, NTNU - Norwegian University of Science and Technology, Trondheim, Norway; 3 Department of Medical Biology, UIT The Artic University of Norway, Tromsø, Norway; 4 Department of Clinical Pathology, University Hospital of North Norway, UNN Tromsø, Norway; 5 Department of Urology, St. Olavs Hospital, Trondheim University Hospital, Trondheim, Norway; 6 Department of Pathology, St. Olavs Hospital, Trondheim University Hospital, Trondheim, Norway; 7 Clinic of Surgery, St. Olavs Hospital, Trondheim University Hospital, Trondheim, Norway

## Abstract

Reactive stroma is a tissue feature commonly observed in the tumor microenvironment of prostate cancer and has previously been associated with more aggressive tumors. The aim of this study was to detect differentially expressed genes and metabolites according to reactive stroma content measured on the exact same prostate cancer tissue sample. Reactive stroma was evaluated using histopathology from 108 fresh frozen prostate cancer samples gathered from 43 patients after prostatectomy (Biobank1). A subset of the samples was analyzed both for metabolic (n = 85) and transcriptomic alterations (n = 78) using high resolution magic angle spinning magnetic resonance spectroscopy (HR-MAS MRS) and RNA microarray, respectively. Recurrence-free survival was assessed in patients with clinical follow-up of minimum five years (n = 38) using biochemical recurrence (BCR) as endpoint. Multivariate metabolomics and gene expression analysis compared low (≤15%) against high reactive stroma content (≥16%). High reactive stroma content was associated with BCR in prostate cancer patients even when accounting for the influence of Grade Group (Cox hazard proportional analysis, p = 0.013). In samples with high reactive stroma content, metabolites and genes linked to immune functions and extracellular matrix (ECM) remodeling were significantly upregulated. Future validation of these findings is important to reveal novel biomarkers and drug targets connected to immune mechanisms and ECM in prostate cancer. The fact that high reactive stroma grading is connected to BCR adds further support for the clinical integration of this histopathological evaluation.

## Introduction

The tumor microenvironment (TME) has in recent years gained attention for its role in cancer cell and tumor development. TME, considered to consist of non-malignant cells and their products, is more genetically stable than cancer cells and supports and allows cancer cells to develop^1,2^. In prostate tumors, TME include activated fibroblasts called cancer associated fibroblasts (CAFs), immune cells and vasculature cells. It is often the site of chronic inflammation and extracellular matrix (ECM) remodeling, similar to what occurs during wound-healing with an increase of activated fibroblasts^2,3^. Such inflammatory TME is usually referred to as ‘reactive stroma’. In prostate cancer, a transition from healthy stroma to reactive stroma has been characterized by a replacement of smooth muscle cells by CAFs and immune cells^3^.

For prostate cancer, the current gold standard for predicting clinical outcome is histopathological evaluation through the Grade Group system^4^. This system sets a grade based on the morphological appearance of prostate glands and cancerous epithelial cells. However, the tumor area can contain clinically relevant histopathologic information that is not captured by the current grading system. Ayala *et al*. were the first to develop a grading system for reactive stroma in prostate cancer and to show that a higher level of reactive stromal response is connected to biochemical recurrence (BCR)^5^. Since then, several studies have linked high reactive stroma content to a worse clinical outcome, including BCR^6–9^, development of castration-resistant prostate cancer^10^ and prostate cancer-specific mortality^11^. In particular, evaluating the tumor stroma was shown to be of extra value in cases were the Grade Group system failed to accurately predict outcome^6^. Although validation and standardization is needed, incorporating reactive stroma into the clinical histopathology evaluation, along with Grade Group, shows potential to optimize prognostic stratification of prostate cancer patients.

As reactive stroma appears to play a significant role in cancer development^12^, it is of interest to understand its underlying molecular mechanisms. These insights may provide new prognostic markers and therapeutic targets. Some molecular features of reactive stroma have already been identified. Smooth muscle differentiation markers such as calponin and desmin are commonly reduced in reactive stroma^3,5,9,10,13^. In contrast, vimentin, pro-collagen and tenascin-C, markers for activated fibroblasts and ECM remodeling, are elevated in reactive stroma in prostate cancer tissue^3,10,13,14^. Reactive stromal cells have also been suggested to promote angiogenesis in the tumor area^15^. Dakova *et al*.^16^ performed global gene expression on laser dissected prostate tissue samples, identifying several differentially expressed genes between reactive and normal stroma. These included genes related to functions such as neurogenesis and DNA repair^16^. Thus, research on proteins and gene expression has revealed changes associated with ECM remodeling, angiogenesis and DNA repair. In contrast, metabolic patterns related to reactive stroma content in prostate cancer tissue are currently unknown. Metabolic reprogramming is a hallmark of cancer and several metabolic alterations has been identified in prostate cancer tissue compared to normal tissue through metabolic profiling, including increase of choline^17^ and sarcosine^18^, and decrease of polyamine and citrate levels^19^.

The aim of our study was to combine histopathology determined reactive stromal grading (RSG) with integrative analysis of metabolomics and transcriptomics data from the same prostate cancer tissue sample, thereby investigating the molecular characteristics of reactive stroma in prostate cancer. Further we investigated how the expression of significant genes and metabolites of reactive stroma are correlated, and investigated biochemical recurrence of patients with high reactive stroma content.

## Methods

### Patients and tissue collection

This study was approved by the Regional committee for Medical and Health Research Ethics (REC) central Norway (identifier 4.2007.1890). All experiments were carried out in accordance to the ethical regulations of REC. All tissue donors signed a written informed consent.

**Figure 1:**
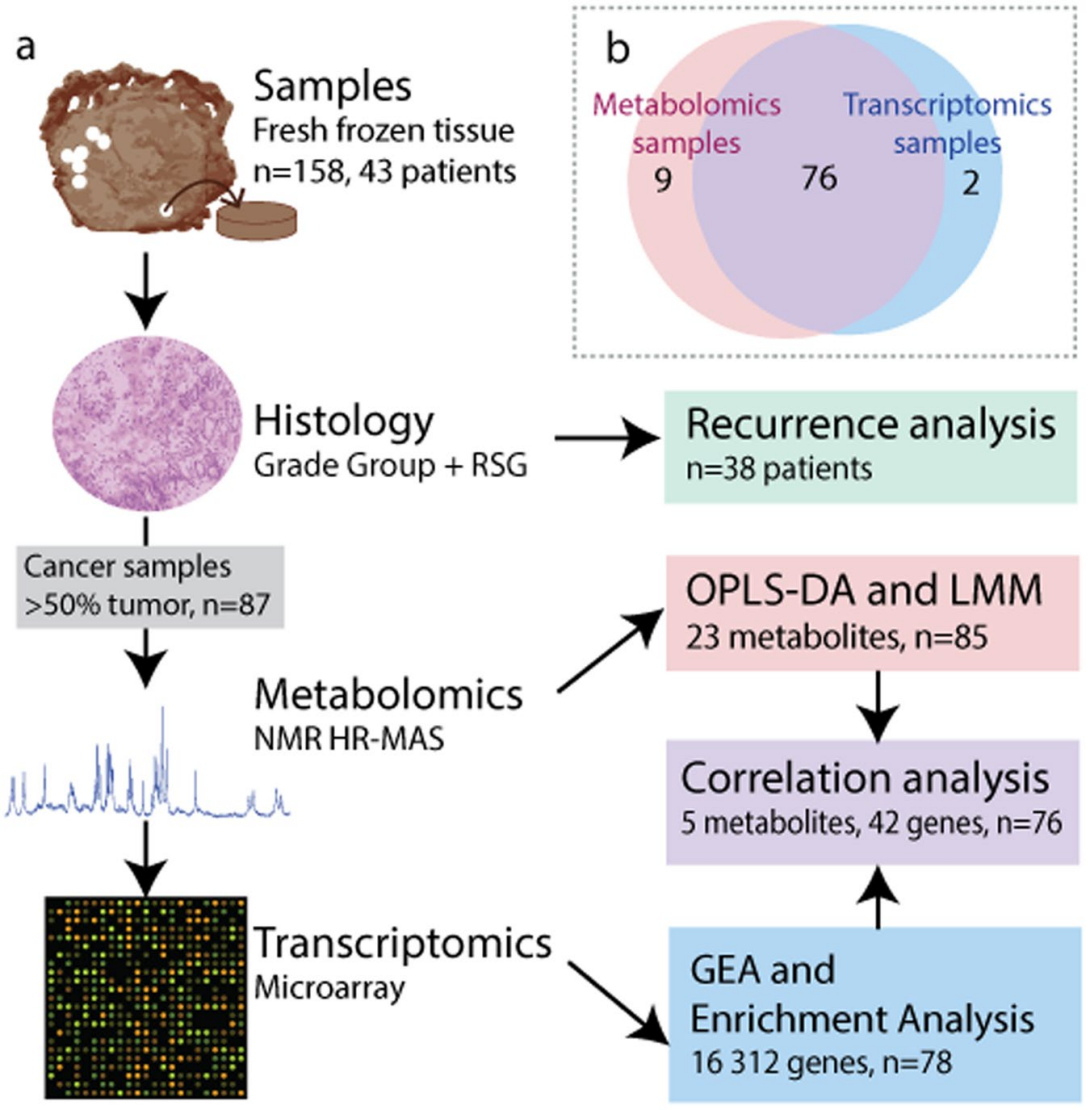
Methodology flowchart. (**a**) Samples were collected from fresh frozen human prostate tissue and cryosections were stained with hematoxylin and eosine. Two pathologists evaluated Grade Group and reactive stroma grade (RSG). Samples with >50% tumor content were selected for further metabolomics and transcriptomics analysis. Data analysis included survival analysis (Kaplan-Meier and Cox hazard proportional analysis) with biochemical recurrence as endpoint, multivariate orthogonal partial least squares discriminant analysis (OPLS-DA), linear mixed models (LMM), gene expression analysis (GEA) and Pearson correlation between selected genes and metabolites. GEA results were used for enrichment analysis. (**b**) Venn diagram of samples used for metabolomics, transcriptomics and both.

Tissue used for this study was donated and collected in 2007 and 2008 ensuing radical prostatectomy. None of the patients received neoadjuvant therapy prior to surgery. A two mm thick tissue slice was cut from the middle of the prostate gland perpendicular to the urethra. The slice was snap frozen in liquid nitrogen on average 15 minutes after surgical removal and stored at −80 °C as previously described by Bertilsson *et al*.^20^. Between four and eleven core tissue samples (three mm diameter) were later collected from each prostate slice (Fig. 1a). In total 158 samples were collected from 43 patients. We obtained at least five years of clinical follow-up from the hospital patient records (Braadland *et al*.^21^) including T-stage, clinical Gleason score (postoperative), tumor volume, preoperative serum prostate specific antigen (PSA) measurements and biochemical recurrence (defined as PSA ≥0.2 ng/ml). The clinical Gleason scores were translated into the new Grade Group system as described by Gordetsky and Epstein^4^.

### Histopathological evaluation

**Figure 2:**
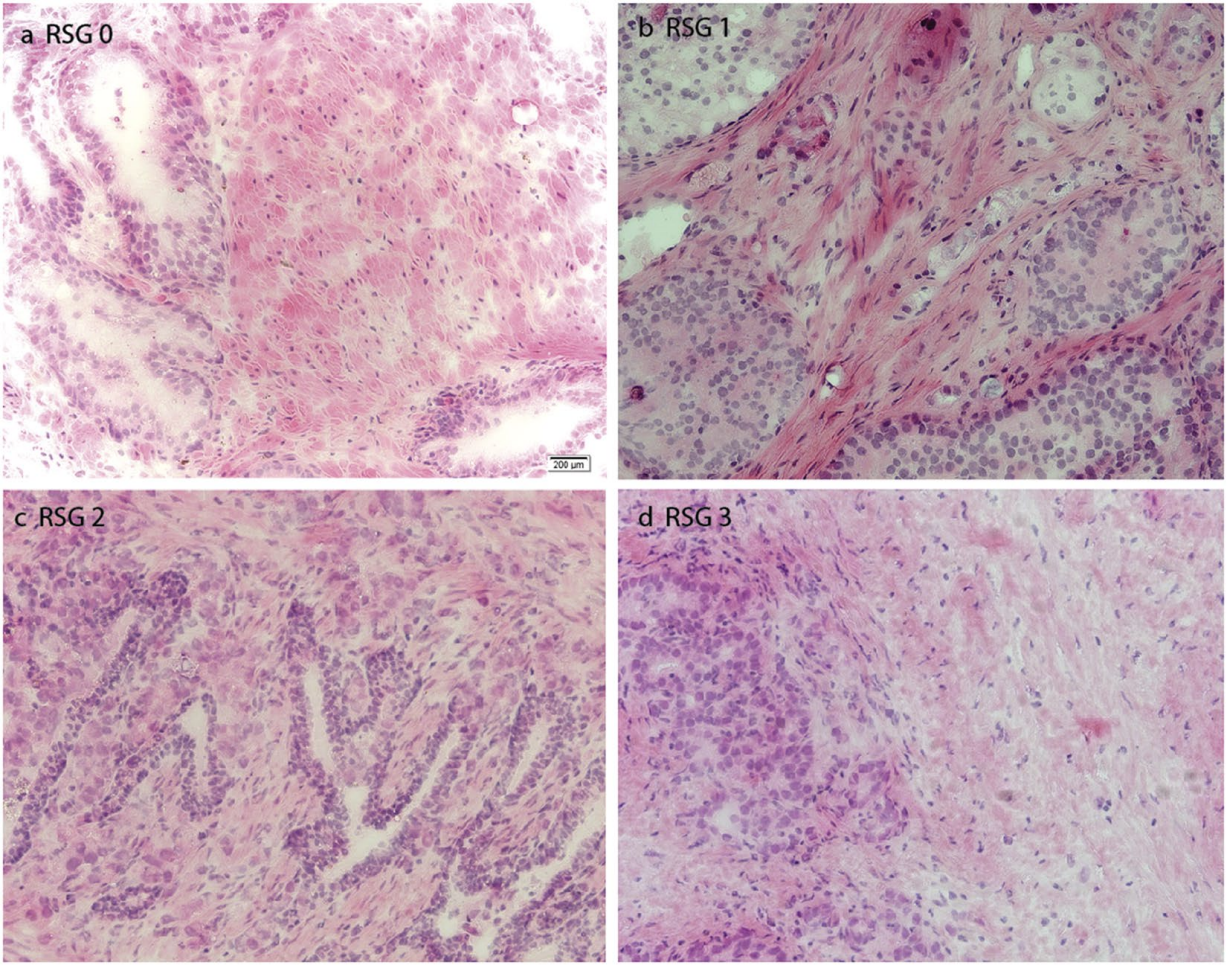
Photomicrographs (x20) of representative hematoxylin and eosinofil stained slides of histopathology of prostate tissue cryosections with reactive stroma grade (RSG) 0–3. (**a**) Normal prostatic tissue with reactive stromal grade (RSG) 0 (<5% reactive stroma). Stroma is mostly consisting of smooth muscle cells making up bundles. (**b**) RSG 1 (6–15% reactive stroma) and Grade Group 4. The majority of stroma still has a strong eosinophilic stain, with a few cells with paler staining appearing, in addition to the presence of more fibroblasts. (**c**) RSG 2 (16–50% reactive stroma) and Grade Group 3. The reactive stroma is more prominent by a weaker eosinophilic stain. (**d**) Sample with RSG 3 (>50% reactive stroma) and Grade Group 3. Here, nearly all normal stroma is replaced by reactive stroma with pale eosinophil staining.

From one side of each fresh frozen tissue sample, a four µm tick cryosection was stained with hematoxylin and eosin (HE). All HE-stained slides (n = 158) were evaluated independently by two experienced uropathologists (E.R. and Ø.S.). Percentage of cancer, normal epithelium and healthy stroma were determined along with Grade Group^4^. Reactive stroma content was defined as the percentage of stroma that was reactive within the tumor area, according to the reactive stroma grade (RSG) system developed by Ayala *et al*.^5^. Each sample was given a grade ranging from 0 to 3: RSG 0 containing 0–5% reactive stroma; RSG 1, 6–15% reactive stroma; RSG 2, 16–50% reactive stroma and RSG 3, 51–100% reactive stroma. Normal prostatic stroma with a high number of smooth muscle cells were characterized by a strong red eosinophilic staining, and the cells by having a large cytoplasm, rounded nuclei and organization into bundles (Fig. 2a). When the stroma gets reactive there will be a replacement of smooth muscle cells by CAFs and immune cells, and the stroma will appear with a paler eosinophilic coloring (Fig. 2b–d). Kappa-statistics was used to calculate a quality score between the two pathologists for both Grade Group and RSG^22^. Later, consensus was reached between the pathologists when there was disagreement on RSG. With disagreement on Grade Group, an independent previous histopathological evaluation by a third pathologist was used to find consensus^20^.

### Metabolomics

Metabolite data was obtained by high-resolution magic angle spinning magnetic resonance spectroscopy (HR-MAS MRS) on fresh frozen tissue samples. HR-MAS MRS spectra were acquired on a Bruker Advance DRX600 (14.1 T) spectrometer (Bruker BioSpin, Germany) with a ^1^H/^13^C MAS probe. LCModel was applied to quantify 23 metabolites from the spectra^23,24^. Further details of the HR-MAS MRS procedure, spectral pre-processing and metabolite quantification are described by Giskeødegård *et al*.^19^. Furthermore, samples containing >50% tumor (n = 85) were selected for molecular and statistical analysis to ensure that the metabolomics profiles mainly represented tumor areas.

### RNA microarray

After HR-MAS MRS, the tissue samples were homogenized and mRNA was extracted. Isolated mRNA was amplified with Illumina TotalPrep RNA amplification Kit (Ambion Inc.) and relative gene expression was subsequently measured with Illumina Human HT-12v4 Expression Bead Chip (Illumina). A comprehensive overview of the protocol and data preprocessing is reported by Bertilsson *et al*.^25^. Here we also selected samples with >50% tumor content (n = 78) for further gene expression analysis (GEA). There was an overlap of 76 samples which were subjected to both metabolite and gene expression analysis (Fig. 1b).

### Multivariate and statistical analysis

Biochemical recurrence (BCR) -free survival analysis included Kaplan Meier and Cox proportional hazards analysis and were performed with the *survival* package in the R environment. BCR was defined as serum PSA >0.2 ng/mL, confirmed by two independent measurements. Time-to-event was set as the number of days between radical prostatectomy and confirmed BCR. Three patients were lost to follow-up and two patients received adjuvant treatment before BCR. As the adjuvant treatment could be influencing the time to BCR, these patients were removed from survival analysis, resulting in a total of 38 patients. As multiple samples were collected from each patient, the sample with the highest RSG was selected as representative for a patient in survival analysis (patient RSG). Patients were divided into a *low RSG* (RSG 0 and 1) group and *high RSG* (RSG 2 and 3) group due to the low numbers of RSG 0 and RSG 3 patients. Covariates included in Cox proportional hazard was *low* vs *high RSG* and clinical Grade Group. For Kaplan-Meier, a log-rank test was used to calculate significance. In addition, to correct for the possible confounding effect of clinical Grade Group and T-stage, a second Kaplan-Meier analysis was performed after removing patients with clinical Grade Group ≥4, as this produced the same median Grade Group and T-stage in both the *low* and *high RSG* group. Pearson correlations between RSG and clinical Grade Group, and RSG and preoperative PSA of the patients were also performed.

Multivariate analysis of the metabolite dataset (23 metabolites, n = 85) was performed in PLSToolbox in the MatLab 8.6.0 (The Mathworks, Inc, USA) environment. The dataset was preprocessed by autoscaling. Supervised orthogonal partial least squares discriminant analysis (OPLS-DA) was used to examine metabolic differences between *high* and *low RSG* using leave-10%-of-patients-out cross-validation and permutation testing for analyzing model reliability (1000 permutations).

Univariate analysis of the 23 quantified log-transformed metabolites was performed with linear mixed models (LMM) in R with the *nlme* package^26^. The relationship between each metabolite concentration and RSG was modeled while correcting for multiple samples per patient. Correct model assumptions were confirmed by qq-plots of model residuals.

Univariate GEA was carried out with the *lumi* and *limma* packages in R for the 23 444 probes, representing 16 312 genes. Samples with *low RSG* were compared to samples with *high RSG*. The result of the GEA was further used to remove duplicated probes so that the dataset only contained one probe per gene. The probe with the lowest adjusted p-value from the GEA was selected for further analysis. The significantly upregulated and downregulated genes were separated into two gene lists, and used for enrichment analysis with Enrichr^27,28^. Results from the background library Gene Ontology (GO) Biological Process 2018 were exported.

Pearson correlation between significant metabolites (n = 5) and the most significantly expressed genes involved in relevant biological processes (n = 42) was calculated in R. Due to lack of normal distribution, the metabolite data was log2-transformed prior to correlation analysis. Five metabolites were selected based on significance in LMM analysis and/or a loading score of ≥±3.0 (first latent variable, OPLS-DA). The genes were selected based on an adjusted p-value < 0.001 from GEA (n = 98, Supplementary Table S1). These genes were manually annotated through genecards.org, and genes with a clear relation to biologically relevant processes were selected for correlation analysis (n = 42).

Unadjusted p-values of ≤0.05 were considered significant for univariate tests and LMM on the metabolic dataset due to a low number of variables (n = 23). Benjamini-Hochberg adjusted p-values ≤ 0.05 were considered significant for GEA, enrichment analysis and gene-metabolite correlations. All confidence intervals (CI) were 95%.

## Results

### Histopathology

A total of 158 samples from 43 patients were histologically evaluated for Grade Group, tumor content and RSG (Fig. 1). Before consensus between pathologists was reached on tumor containing samples (n = 108), the original evaluations gave a kappa score of 0.64 and 0.30 for Grade Group and RSG, respectively. An overview of histopathology and clinical data are listed in Table 1.The majority of samples (n = 48, 55.2%) and patients (n = 24, 63.2%) were scored as RSG 1, while the least prevalent score was RSG 3 with four samples (4.6%) and two patients (5.3%). There was a clear correlation between clinical Grade Group and RSG of patients (R = 0.56, ${\rm{p}}=0.23\ast {10}^{-3}$). There was also a weak, but significant, correlation between RSG and Grade Group in the samples (R = 0.25, p = 0.018). There was no correlation between preoperative PSA levels and patient RSG (R = 0.015, p = 0.93).

**Table 1:** Histology of samples and clinical data of patients.

	RSG 0	RSG 1	RSG 2	RSG 3	Total
**Samples with >50% tumor used for metabolomics (n = 85)**					
Samples (percent)	11 (12.9%)	47 (55.3%)	23 (27.1%)	4 (4.7%)	85
Median Grade Group (range)	3 (1–5)	1 (1–5)	3 (1–5)	4.5 (3–5)	2 (1–5)
Mean tumor percent (range)	89.5 (70–100)	82.3 (60–92.5)	83.2 (62.5–100)	88.1 (72.5–97.5)	83.6 (60–100)
**Samples with >50% tumor used for transcriptomics (n = 78)**					
Samples (percent)	10 (12.8%)	41 (52.3%)	23 (29.5%)	4 (5.2%)	78
Median Grade Group (range)	2.5 (1–5)	1 (1–5)	3 (1–4)	4.5 (3–5)	2 (6–10)
Mean tumor percent (range)	87.5 (70–100)	83.2 (57.5–95)	83.2 (62.5–100)	88.1 (72.5–97.5)	84.0 (57.5–100)
**Clinical variables of Patients (n = 38)**					
Patients (percent)	2 (5.3%)	24 (63.2%)	10 (26.3%)	2 (5.3%)	38
Recurrence, 5 year follow-up (percent)	0	1 (4.2%)	7 (70.0%)	1 (50.0%)	11 (28.9%)
Mean age at operation (range)	58.5 (56–61)	61.4 (48–69)	62.1 (48–68)	68.5 (68–69)	61.8 (48–69)
Median Grade Group (range)	2 (2)	2 (1–5)	3.5 (1–5)	5 (5)	3 (1–5)
Median pathological stage (range)	T2c (T2c)	T2c (T2a–T3b)	T3a (T2c–T3b)	T3b (T3a–T3b)	T2c (T2a–T3b)
Mean preoperative serum PSA (range)	8.0 (5.2–10.7)	10.8 (3.7–45.8)	10.6 (5.2–17.0)	9.75 (5.6–13.9)	10.3 (3.7–48.8)

RSG = reactive stroma grade, PSA = prostate specific antigen.

### *High RSG* predict shorter BCR-free survival independent of Grade Group

**Figure 3:**
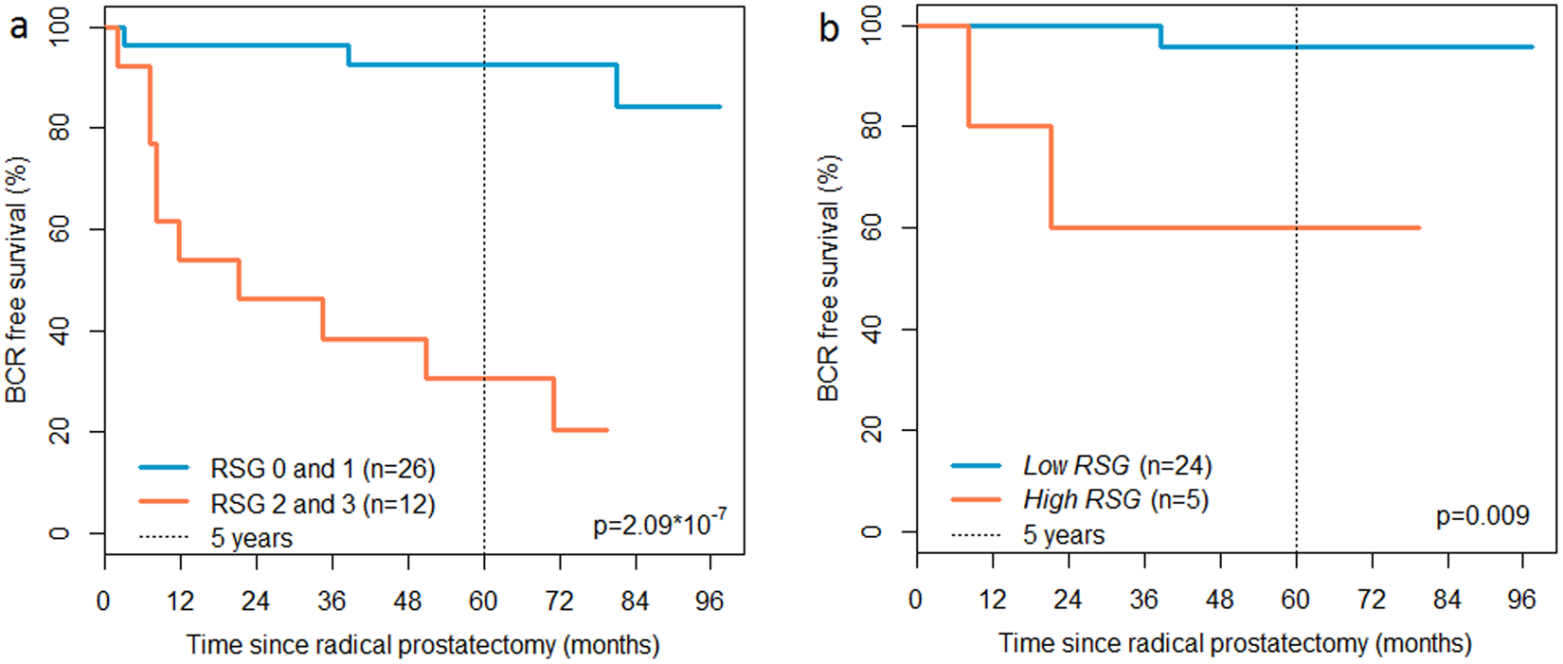
Kaplan-Meier plots of biochemical recurrence (BCR). Kaplan-Meier analysis were performed on (**a**) all patients (n = 38) and (**b**) patients with low-to-medium Grade Group (≤3) (n = 29).

A total of 38 patients had sufficient clinical follow-up data and were included in survival analysis where *low RSG* (n = 26) was compared to *high RSG* (n = 12). Kaplan-Meier analysis showed significantly better BCR-free survival in patients with *low RSG*, having 92.3% recurrence-free survival, and *high RSG* patients having 25.0% recurrence-free survival after 5 years of follow-up (${\rm{p}}=2.09\ast {10}^{-7}$) (Fig. 3a). However, the *low* and *high RSG* patient groups had a different median clinical Grade Group of 2 and 4, respectively (two-sided t-test, p = 0.013). In addition, these two groups also had a significant different median T-stage of T2c for *low RSG* and T3a for *high RSG* (two-sided t-test, ${\rm{p}}=0.16\ast {10}^{-3}$). A second Kaplan-Meier analysis was therefore performed for patients with Grade Group ≤3, resulting in a total of 29 patients. This second selection of *low* (n = 24) and *high RSG* (n = 5) patients had the same median Grade Group of 2 and median T-stage of T2c and still displayed a significant recurrence-free 5-year survival difference (BCR-free survival 95.8% for *low RSG* and 60% for *high RSG*, p = 0.009) (Fig. 3b). Multivariate Cox proportional hazard model of all 38 patients provided hazard ratios of 16.44 (p = 0.013, CI = 1.81–149.20) for RSG and 1.95 (p = 0.018, CI = 1.12–3.40) for Grade Group.

### Reactive stroma shows metabolic alteration

**Figure 4:**
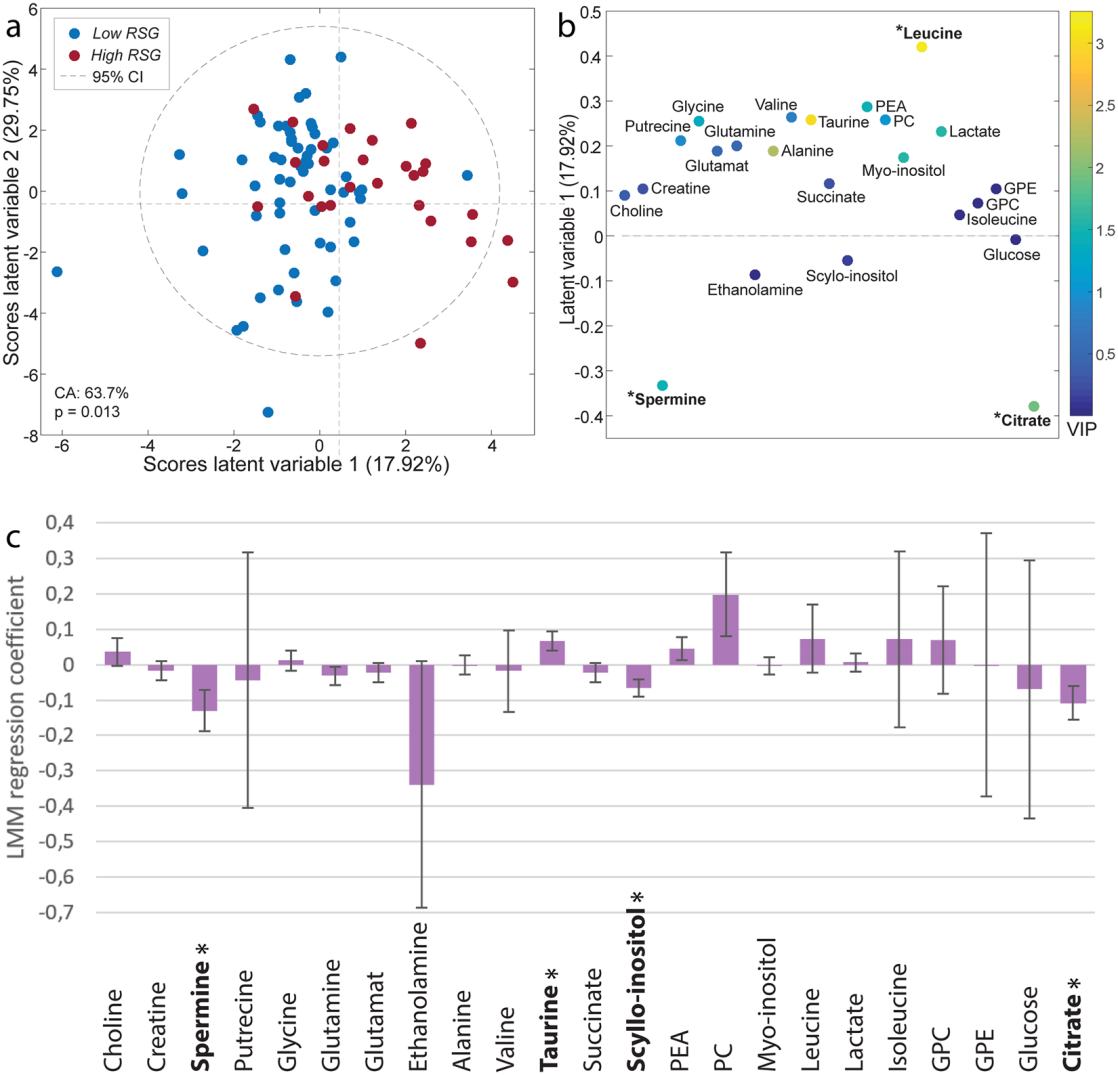
Metabolite analysis in samples with *high* and *low* reactive stroma grading (RSG). (**a**) Scores plot and (**b**) loadings plot from OPLS-DA model where *low RSG* (RSG 0 and 1, n = 58) were compared to *high RSG* (RSG 2 and 3, n = 27). Variables in the loadings plot are color-coded by variable importance in the projection (VIP), which is an estimate of each variables contribution to the model. Metabolites with a loadings score ≥3.0 or ≤−3.0 are indicated by * (**c**) Univariate linear mixed model (LMM) regression coefficients with increasing RSG. Error bars represent standard error and significant metabolites are indicated by *. Abbreviations: CA = classification accuracy, GPC = Glycerophosphocholine, GPE = Glycerophosphoethanol, PC = phosphocholine, PEA = phosohpoethanolamine and GPE = Glycerophosphoethanol.

Multivariate OPLS-DA analysis using quantified values for 23 metabolites showed a significant difference between *high* and *low RSG* (p = 0.014, accuracy 64.9%, sensitivity 75.0% and specificity 54.9%, Fig. 4a,b). The loadings depicted in Fig. 4b show that there are lower levels of citrate and spermine and higher levels of leucine in samples with *high RSG*.

Univariate LMM testing of each quantified metabolite modeled against RSG values 0–3 resulted in four significant metabolites (Fig. 4c). Taurine (p = 0.018) was found at elevated levels, while citrate (p = 0.027), spermine (p = 0.031) and scyllo-inositol (p = 0.009) were found at lower levels with increasing RSG.

### Genes involved in immune responses and ECM remodeling are upregulated in reactive stroma

**Figure 5:**
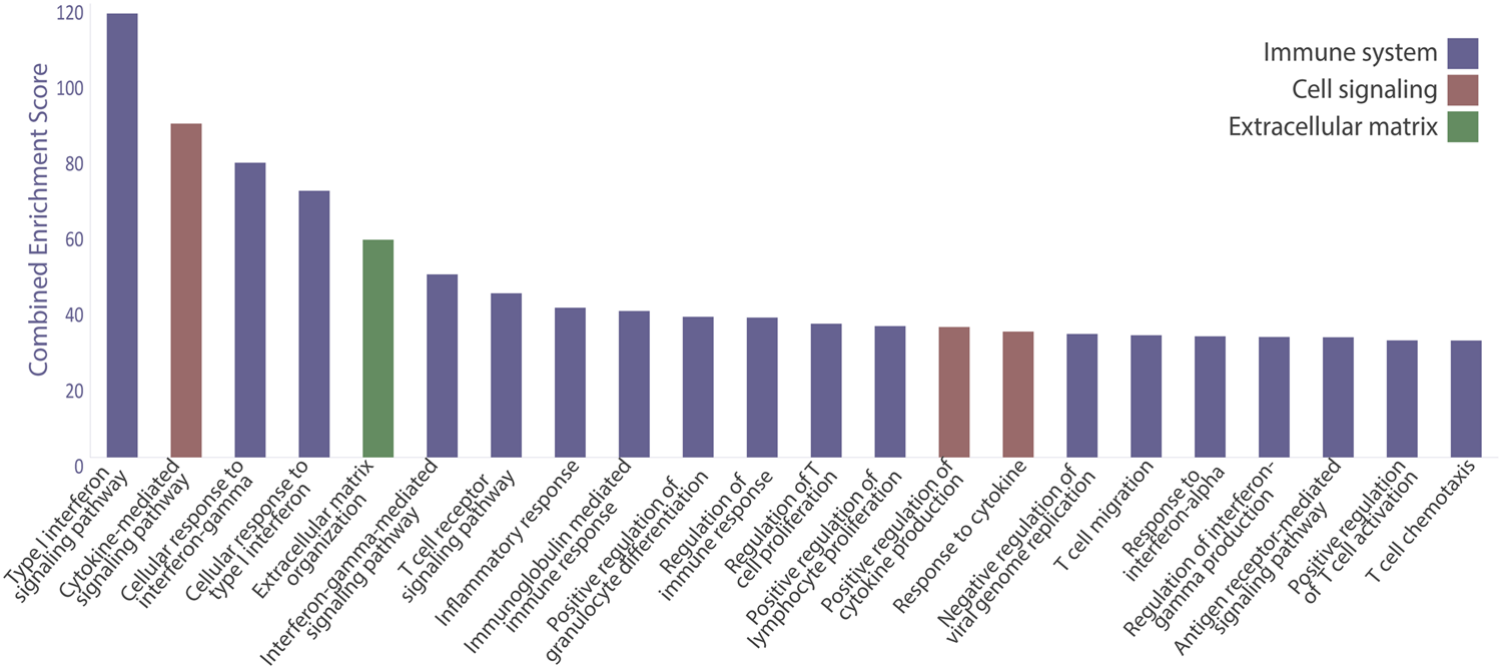
Enrichment analysis of Gene Ontology (GO) Biological Processes using Enrichr. Analysis was based on output from gene expression analysis (GEA) comparing *low RSG* (RSG 0 and 1) to *high RSG* (RSG 2 and 3). The figure includes all biological process terms with a combined enrichment score over 30.

Gene expression analysis (GEA) was performed comparing *high RSG* to *low RSG*. A total of 609 and 471 genes were up- and downregulated, respectively. Enrichment analysis was performed with Enrichr using gene lists of significantly up- and downregulated genes, which produced 339 significantly upregulated and seven significantly downregulated enriched biological process terms in *high* compared to *low RSG* (Supplementary Table S2). All biological terms with a combined score (calculated by Enrichr) over 30 are presented in Fig. 5. Of these terms (n = 22), all were upregulated and 18 were related to the immune system, three to cell signaling and one was related to extracellular matrix.

### Correlation between selected genes and metabolites

**Figure 6:**
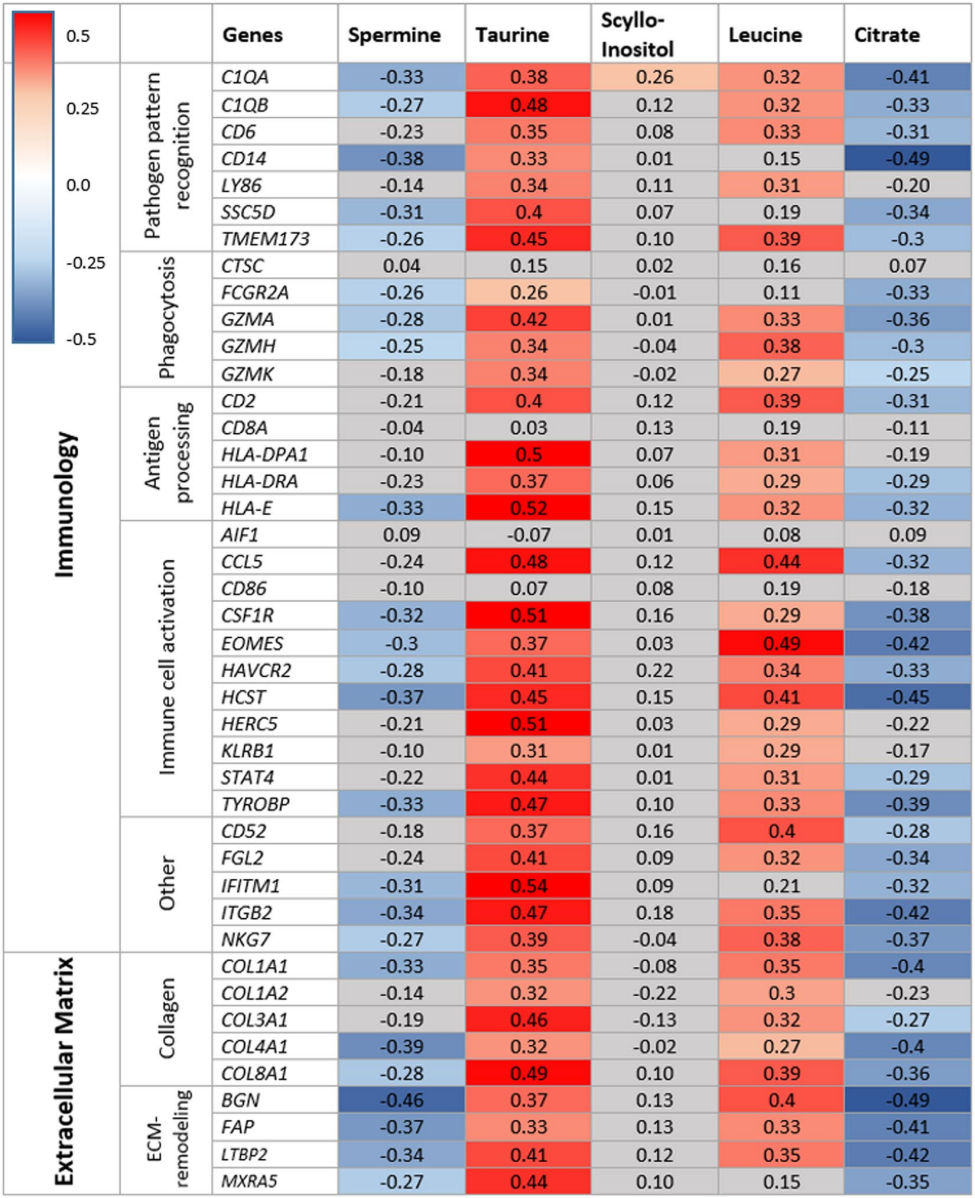
Correlation analysis between selected metabolites and genes. Values are Pearson correlation coefficients. Values marked with red (positive correlation) or blue color (negative correlation) were significant after Benjamini-Hochberg adjustment, while values marked with light grey were non-significant. Color intensity corresponds to the correlation coefficient value.

A total of 42 upregulated genes and five metabolites (spermine, taurine, scyllo-inositol, leucine and citrate) were selected for correlation analysis. Immunology and ECM were considered relevant biological processes to reactive stroma based on our enrichment results and the literature^29,30^, and were along with level of significance, used as selection criteria for the genes. Nine genes were related to ECM and 33 were related to immunology, which could be further categorized into various different functions of the immune system and ECM (Fig. 6). Of the selected genes, four immunology-related genes, *CTSC*, *AIF1*, *CD8A* and *CD86*, were not correlated with any of the metabolites. Taurine was correlated with all the remaining 38 genes, while scyllo-inostol only correlated with one gene, *C1QA*. Citrate and spermine were negatively correlated to all genes, while taurine, scyllo-inositol and leucine were positively correlated with all genes that had a significant correlation.

## Discussion

In this study we have demonstrated that reactive stroma content in human prostate cancer tissue is associated with metabolic and transcriptomic alteration, and significantly influence biochemical recurrence. The gene expression analysis showed that reactive stroma is clearly connected to inflammatory responses, one of the triggers of cancer initiation^31^. Our results suggest that grading of reactive stroma could be a valuable supplement to Grade Group.

Although the Grade Group system is currently the gold standard for assessing patient prognosis and aggressiveness of prostate cancer, there is still a need for improvements especially within Grade Group 2 and 3^29^. Histological reactive stroma grading could provide further strength to the Grade Group evaluation and improve patient prognosis assessment^6–9,11^. In our study, both the Kaplan-Meier and the Cox analysis showed a significantly worse BCR-free survival in patients with a *high* compared to *low RSG*, even when accounting for Grade Group (p = 0.009 and p = 0.013, respectively) (Fig. 3).

This indicates that reactive stroma content can provide additional information beyond the Grade Group system used for current patient prognosis assessment in prostate cancer, which is in line with previous studies^6–9,11^. However, RSG is not entirely independent of Grade Group, as illustrated by our correlation analysis and previous studies^7,11,14^. This suggest that a reactive stromal response is coevolving with cancer aggressiveness and supporting tumor progression.

Our statistical analysis of 23 different tissue metabolites indicated that the levels of spermine, citrate, taurine, leucine and scyllo-inostol were different between *low* and *high RSG* (Fig. 4). Since RSG and Grade Group are correlated with each other, it is not possible to robustly assess whether the changes in these metabolites are due to reactive stroma or Grade Group. Spermine and citrate are normally found at high levels in healthy prostate tissue compared to other human tissues, as these metabolites are secreted by the gland^32^. We have previously shown lower spermine and citrate levels to be predictors of aggressive cancer^19,21^. Reduced citrate and spermine levels have not previously been connected to typical processes involved in reactive stroma, such as inflammation and ECM remodeling as presented in this study. It is therefore possible that the reduced levels of citrate and spermine are a result of tumor cell growth rather than *high RSG*, and further studies are necessary to assess the connection to inflammation and ECM remodeling.

Citrate and spermine concentrations were negatively correlated with selected genes (selected based on significance level in GEA), while leucine and taurine concentrations were positively correlated with the selected genes (Fig. 6). A high number of significant correlations are to be expected since both the genes and the metabolites were selected based on analysis which compared *low RSG* to *high RSG*. However, our analysis reveals in which direction these metabolites and genes interact. Interestingly, taurine was significantly positively correlated with the highest number of genes (38 of 42). Taurine is known to be a prevalent metabolite in most tissues and one of its functions is protection against oxidative stress produced by inflammatory reactions^33^. In prostate cancer, an elevation of taurine levels compared to healthy tissue is reported^34,35^, but no significant difference in taurine has been found in this cohort, neither between cancer and non-cancer tissue nor between low and high Grade Group prostate cancer^19^. Significantly higher levels of taurine in reactive stroma (p = 0.018) suggest that elevated taurine levels may be a response to inflammation in reactive stroma. Scyllo-inostol concentrations were significantly elevated in *high RSG* compared to *low RSG* in univariate (p = 0.009), but not multivariate analysis. Although previously found to be elevated in prostate cancer^34^, no biological process has been suggested and its potential role in prostate cancer remains unclear. The amino acid leucine is another metabolite found at higher levels in *high RSG* samples (Fig. 4b). Leucine is a key amino acid of proteoglycans such as decorin and biglycan. These molecules function as building blocks during ECM remodeling and are found with elevated expression in tumor stroma^36^. Biglycan expression was upregulated among *high RSG* samples in this study (${\rm{p}}=1.23\ast {10}^{-4}$) and is previously reported to attract pro-inflammatory macrophages in both cell culture and mice^37^. In sum, our metabolic profile appears to be linked to inflammation and ECM remodeling.

The results from gene enrichment analysis indicated that genes involved in immunity, cell signaling and extra cellular matrix were particularly important when comparing *low* and *high RSG* (Fig. 5). Cellular signaling pathways are known to be reprogrammed in cancer cells^38^ and our results from the enrichment analysis may therefore represent both cancer cell and the cross-talk between cancer cells and reactive stroma. One known example is transforming growth factor-β (TGF-β), significantly upregulated in our GEA (p = 0.003, Supplementary Data S1), which is secreted by cancer cells, activates fibroblasts and promotes ECM remodeling^39^. Remodeling of the ECM is, together with inflammation, a feature of the reactive stroma^40^, and is parallel to chronic wound repair.

Among the genes which were selected based on level of significance between *low* and *high RSG* and their involvement in immunity and ECM remodeling, we found 12 genes that were specifically involved in pathogen responses, such as phagocytosis, pathogen pattern recognition and antigen processing (Fig. 6). Additionally, biological processes related to interferon signaling were particularly enriched (Fig. 5). Interferons are a group of signaling proteins which are secreted from cells as a response to pathogen infections^41^. Infectious pathogens like bacteria and viruses may be involved in chronic inflammation and further progression of cancer^42^. In previous studies different pathogens were correlated with prostate cancer initiation, including high risk human papilloma virus (HR-HPV)^43^, *Enterobacteriaceae* species^44^ and *Porpionibacterium acnes*^44–47^. In our study, both the genes *CD6* and *CD14*, which are directly involved in recognition of surface bound bacterial lipopolysaccharide (LPS)^48,49^, were expressed higher in *high RSG* samples. The fact that genes specifically involved in both recognition and destruction of pathogens are among the most highly expressed genes in *high RSG*, suggest the presence of infectious agents contributing to the reactive stromal response. Future studies using sensitive methods suitable for detecting suspected pathogens are needed.

Several genes involved in immune cell activation were differentially expressed between *high* and *low RSG* (Fig. 6). Many of these genes are involved in regulation of inflammatory responses, by modifying the functions of T-cells, macrophages and natural killer cells, and can either be pro-inflammatory or inhibit immune responses. These genes include *CCL5* and *CSF1R*. CCL5 is a pro-inflammatory chemokine that attracts immune cells such as macrophages, T-leukocytes, eosinophils and basophiles^50^. CCL5 has previously been linked to cancer progression in prostate^51^. CSF1R is a pro-inflammatory receptor mainly found on macrophages and monocytes. It is thought to trigger recruitment, growth and proliferation of these cells in cancer, and blocking this receptor was found to suppress tumor growth in combination with irradiation therapy in prostate cancer patients^52^. These data indicate that the tissue is inflamed by the actions of an array of immune cells.

Several genes related to remodeling of the ECM were upregulated in reactive stroma in this study (Fig. 6). One of the key contributors to reactive stroma is a group of activated fibroblasts, CAFs. The function of these cells is to remodel the ECM^53^. CAFs have an elevated production of α smooth muscle actin (α-SMA) and fibroblast activation protein (FAP) compared to other cells in the tissue^53,54^. *FAP* is selectively expressed by activated fibroblasts during either wound-healing responses or by CAFs in epithelial cancers^55,56^, and was found to have increased expression in *high RSG* in our cohort (p = 0.001). Expression of α-SMA is also a key characteristic of CAFs, but it was not found to be differentially expressed in reactive stroma of this study (p = 0.34). A possible explanation for this observation is that α-SMA is also produced by smooth muscle cells^40^, so any increase in fibroblast-derived α-SMA may be hidden by a reduction of smooth muscle-derived α-SMA.

Collagen is the most abundant type of protein making up the ECM, and various collagen genes had increased expression in *high RSG* samples in our study. In cancer, breakdown and re-deposition of collagen is common and causes cancer progression through destabilization of cell polarity and cell-to-cell adhesion^57^. Collagen building is thought to be partly organized by the proteoglycan biglycan^58^. Biglycan is encoded by the gene *BGN*, which was higher expressed in *high RSG* (${\rm{p}}=0.12\ast {10}^{-3}$). Up-regulation of *BGN* has previously been linked to poor prognosis in prostate cancer^59^. Another proteoglycan encoding gene which were higher expressed in *high RSG*, *MXRA5*, has a similar function to *BGN* and is associated with several forms of cancers^60^. These findings reflect the remodeling of ECM which occurs in reactive stroma, and suggest that a higher number of CAFs are likely present due to the high expression of *FAB*, a selective marker for activated fibroblasts.

Even though stromal grading shows clinical potential, RSG evaluation will still need standardization before it can be implemented in the clinic, clearly indicated by the kappa score for RSG (κ = 0.30) which was considerably lower than for Grade Group (κ = 0.64). To our knowledge, no kappa score was included in any of the previous published studies, and it is therefore not possible to compare the robustness of our evaluation to others. Progress are being made to optimize characterization of reactive stroma^61^ and there is a need to quality check and quantify the variation between individual pathologists. In addition, evaluating RSG on cryosections caused further limitation in this study due to common lower staining quality compared to sections from formalin fixed paraffin embedded tissue. There is higher requirement for section quality and staining when assessing RSG compared to assessing Grade Group.

In this study we have demonstrated that reactive stroma grading of prostate cancer offer additional prognostic value as a supplement to the clinical Grade Group assessment. However, for applying RSG in the routine clinical assessment, more standardized scoring criteria is needed. Metabolic and translational differences between samples with *high* and *low* reactive stroma content were also identified. In particular, genes related to immunology and ECM remodeling were upregulated in samples with high reactive stroma content. Molecular understanding of the reactive stroma may lead to new diagnostic and therapeutic tools. Identifying therapeutic targets residing in reactive stroma, could be of particular benefit due to the higher degree of genetic stability compared to cancer cells. Hence, such therapeutic targets might be less prone to treatment resistance.

## Electronic supplementary material

Supplementary Information

Dataset S1
