# Supplementary material for: Integrative metabolic and transcriptomic profiling of prostate cancer tissue containing reactive stroma
Source: Sci Rep. 2018 Sep 24;8:14269. doi: 10.1038/s41598-018-32549-1 (PMC6155140; doi:10.1038/s41598-018-32549-1)
Supplement: Supplementary file 1 — Supplementary Information [file 41598_2018_32549_MOESM1_ESM.pdf]

Supplementary to:

Integrative metabolic and transcriptomic profiling of prostate cancer tissue containing reactive stroma

Maria K. Andersen[1], Kjersti Rise[2], Guro F. Giskeødegård[1], Elin Richardsen[3,4], Helena Bertilsson[2,5], Øystein Størkersen[6], Tone F. Bathen[1], Morten Rye[2,7], May-Britt Tessem[1]**

[1]Department of Circulation and Medical Imaging, NTNU - Norwegian University of Science and Technology, PO Box 8905 MTFS, Trondheim NO-7491, Norway.

[2] Department of Clinical and Molecular Medicine, NTNU - Norwegian University of Science and Technology, PO Box 8905 MTFS, Trondheim NO-7491, Norway

[3] Department of Medical Biology, UIT The Artic University of Norway, PO Box NO-6050, 9037 Tromsø, Norway.

[4] Department of Clinical Pathology, University Hospital of North Norway, NO-9038, UNN Tromsø, Norway.

[5] Department of Urology, St. Olavs Hospital, Trondheim University Hospital, PO Box 3250, NO-7006 Trondheim, Norway.

[6] Department of Pathology, St. Olavs Hospital, Trondheim University Hospital, PO Box 3250, NO-7006 Trondheim, Norway.

[7] Clinic of Surgery, St. Olavs Hospital, Trondheim University Hospital, PO Box 3250, NO-7006 Trondheim, Norway.

Correspondence:

*May-Britt Tessem and Maria K. Andersen, Department of Circulation and Medical Imaging, Faculty of Medicine and Health Sciences, NTNU, PO Box 8905, NO-7491 Trondheim, Norway.

Email: may-britt.tessem@ntnu.no, maria.k.andersen@ntnu.no

Supplementary Table S1: All genes with a Benjamin-Hochberg adjusted p-value < $1*10^{-3}$ after gene expression analysis (GEA). Positive log fold change indicates increase of expression in *high RSG* (RSG 2 and 3). Full result of GEA is accessible in Supplementary Data S1.

Gene ID	GeneCard ID	Log Fold-Change	Adjusted P-value	Gene ID	GeneCard ID	Log Fold-Change	Adjusted P-value
CD8A	GC02M086784	0.81	$8.48*10^{-6}$	NREP	GC05M111662	0.58	$3.83*10^{-4}$
CCL5	GC17M035871	0.93	$1.27*10^{-5}$	PCCA	GC13P100089	-0.41	$3.83*10^{-4}$
CTSC	GC11M088211	0.64	$4.02*10^{-5}$	SLCO2B1	GC11P075320	0.57	$4.48*10^{-4}$
EVI2B	GC17M031302	0.58	$4.02*10^{-5}$	ABHD10	GC03P111978	-0.43	$4.79*10^{-4}$
FGL2	GC07M077193	0.71	$4.02*10^{-5}$	SLC35E1	GC03P111978	-0.29	$4.91*10^{-4}$
GZMH	GC14M024606	0.62	$4.02*10^{-5}$	CSF1R	GC05M150053	0.63	$5.09*10^{-4}$
GZMK	GC05P055024	0.84	$4.02*10^{-5}$	GIMAP7	GC07P150514	0.61	$5.32*10^{-4}$
TMEM173	GC05M139475	0.55	$9.26*10^{-5}$	GZMA	GC05P055102	0.90	$5.32*10^{-4}$
TYROBP	GC19M035904	0.82	$9.64*10^{-5}$	PATL2	GC15M044665	0.39	$5.32*10^{-4}$
CYTH4	GC22P037282	0.49	$1.22*10^{-4}$	RNASE6	GC14P020781	0.45	$5.32*10^{-4}$
BGN	GC0XP153494	0.76	$1.23*10^{-4}$	KLRB1	GC12M011717	0.45	$5.71*10^{-4}$
ARHGAP30	GC01M161016	0.42	$1.28*10^{-4}$	PRRX1	GC01P170662	0.67	$5.71*10^{-4}$
CBS	GC21M043053	-0.89	$1.28*10^{-4}$	RGS1	GC01P192544	0.88	$5.71*10^{-4}$
GIMAP4	GC07P150568	0.63	$1.28*10^{-4}$	MS4A7	GC11P060396	0.46	$5.80*10^{-4}$
GNE	GC09M036214	-0.91	$1.28*10^{-4}$	SAMD9L	GC07M093130	0.51	$5.80*10^{-4}$
HLA-E	GC06P031055	0.74	$1.28*10^{-4}$	CREB3L4	GC01P153967	-0.56	$6.48*10^{-4}$
ITGB2	GC21M044885	0.87	$1.28*10^{-4}$	COL3A1	GC02P188974	0.93	$6.54*10^{-4}$
PARP1	GC01M226360	0.33	$1.28*10^{-4}$	LRTOMT	GC11P072080	-0.34	$6.54*10^{-4}$
COL1A1	GC17M050183	1.04	$1.36*10^{-4}$	C1QB	GC01P022652	0.90	$6.69*10^{-4}$
FAP	GC05P112707	0.45	$1.36*10^{-4}$	LOC643733	GC11M104901	0.39	$6.69*10^{-4}$
AIF1	GC06P031908	0.75	$1.40*10^{-4}$	FCGR2A	GC01P161505	0.37	$6.70*10^{-4}$
RGS10	GC10M119499	0.34	$1.63*10^{-4}$	AZGP1	GC07M099967	-0.66	$6.74*10^{-4}$
CD14	GC05M140594	0.63	$1.67*10^{-4}$	NRCAM	GC07M108147	-0.86	$6.74*10^{-4}$
SLC23A1	GC05M139377	-0.72	$1.67*10^{-4}$	EOMES	GC03M027715	0.90	$6.77*10^{-4}$
CD86	GC03P122055	0.40	$1.89*10^{-4}$	PHLDB1	GC11P118606	0.41	$6.77*10^{-4}$
NKG7	GC19M051371	0.61	$2.10*10^{-4}$	RCSD1	GC01P167599	0.42	$7.13*10^{-4}$
HERC5	GC04P088457	0.58	$2.11*10^{-4}$	KIF13B	GC08M029067	-0.57	$7.49*10^{-4}$
COL1A2	GC07P094394	0.81	$2.55*10^{-4}$	MXRA5	GC0XM003308	0.73	$7.49*10^{-4}$
EIF2AK3	GC02M088637	-0.44	$2.58*10^{-4}$	CYP2J2	GC01M059893	-0.82	$7.50*10^{-4}$
SHANK2	GC11M070467	-0.63	$2.58*10^{-4}$	METTL9	GC16P021610	-0.31	$7.54*10^{-4}$
PXMP4	GC20M033706	-0.53	$2.59*10^{-4}$	SAMD3	GC06M130144	0.55	$7.54*10^{-4}$
ALG8	GC11M078100	-0.35	$2.88*10^{-4}$	NOL3	GC16P067207	-0.58	$8.03*10^{-4}$
CD2	GC01P116754	0.68	$2.88*10^{-4}$	HLA-DPA1	GC06M033032	0.79	$8.69*10^{-4}$
DHTKD1	GC10P012068	-0.32	$3.08*10^{-4}$	STAT4	GC02M191029	0.44	$8.72*10^{-4}$
COX15	GC10M099696	-0.40	$3.50*10^{-4}$	HAVCR2	GC05M157063	0.48	$8.89*10^{-4}$
RPL28	GC19P055388	-0.62	$3.52*10^{-4}$	KIAA0319L	GC01M035433	-0.39	$8.89*10^{-4}$
LY86	GC06P006588	0.40	$3.70*10^{-4}$	CORO1A	GC16P030194	0.49	$9.16*10^{-4}$
COL4A1	GC13M110148	0.61	$3.79*10^{-4}$	C1QA	GC01P022636	0.52	$9.38*10^{-4}$
COL8A1	GC03P099638	1.10	$3.79*10^{-4}$	CCDC109B	GC04P109561	0.50	$9.38*10^{-4}$
PDGFRB	GC05M150113	0.58	$3.79*10^{-4}$	CD6	GC11P060990	0.79	$9.38*10^{-4}$
ASAP1	GC08M130052	0.37	$3.83*10^{-4}$	DOCK10	GC02M224765	0.55	$9.38*10^{-4}$
CD52	GC01P026317	0.79	$3.83*10^{-4}$	LTBP2	GC14M074498	0.68	$9.38*10^{-4}$
EPSTI1	GC13M042886	1.07	$3.83*10^{-4}$	PCA3	GC09P076764	-1.77	$9.38*10^{-4}$
GBP4	GC01M089181	0.78	$3.83*10^{-4}$	PNPLA4	GC0XM007826	-0.47	$9.38*10^{-4}$
HCST	GC19P036426	0.68	$3.83*10^{-4}$	RASSF5	GC01P206507	0.34	$9.38*10^{-4}$
HLA-DRA	GC06P032412	0.87	$3.83*10^{-4}$	SNX7	GC01P098590	0.27	$9.38*10^{-4}$
HLA-DRB6	GC06M032555	0.84	$3.83*10^{-4}$	SSC5D	GC19P055488	0.59	$9.38*10^{-4}$
IFITM1	GC11P000313	0.95	$3.83*10^{-4}$	THBS2	GC06M169215	0.85	$9.38*10^{-4}$
MS4A6A	GC11M060189	0.73	$3.83*10^{-4}$	TRPM8	GC02P233917	-1.04	$9.38*10^{-4}$

Supplementary Table S2: Result of gene set enrichment analysis from Enrichr, using gene ontology (GO) biological process gene sets. Combined scores are calculated from the p-values and z-scores through the equation ln(p) * z.

Analysis based on significantly up-regulated genes						
GO term	GO ID	Overlap	P-value	Adjusted P-value	Z-score	Combined Score
Type I interferon signaling pathway	GO:0060337	26/66	1.17E-22	1.06E-19	-2.32	117.34
Cytokine-mediated signaling pathway	GO:0019221	82/634	3.38E-29	9.21E-26	-1.35	88.21
Cellular response to interferon-gamma	GO:0071346	28/117	1.18E-17	8.01E-15	-2.00	77.86
Cellular response to type I interferon	GO:0071357	26/66	1.17E-22	1.06E-19	-1.39	70.41
Extracellular matrix organization	GO:0030198	36/230	5.86E-16	2.28E-13	-1.64	57.46
Interferon-gamma-mediated signaling pathway	GO:0060333	22/71	9.24E-17	5.04E-14	-1.31	48.28
T cell receptor signaling pathway	GO:0050852	31/164	2.81E-16	1.28E-13	-1.21	43.28
Inflammatory response	GO:0006954	30/253	2.16E-10	4.20E-08	-1.77	39.49
Immunoglobulin mediated immune response	GO:0016064	5/9	2.93E-06	1.67E-04	-3.03	38.60
Positive regulation of granulocyte differentiation	GO:0030854	4/7	2.77E-05	8.56E-04	-3.53	37.07
Regulation of immune response	GO:0050776	34/252	3.30E-13	9.99E-11	-1.28	36.87
Regulation of T cell proliferation	GO:0042129	14/63	4.80E-09	6.54E-07	-1.84	35.24
Positive regulation of lymphocyte proliferation	GO:0050671	17/72	3.81E-11	8.00E-09	-1.44	34.61
Positive regulation of cytokine production	GO:0001819	25/221	1.82E-08	2.15E-06	-1.93	34.38
Response to cytokine	GO:0034097	24/139	5.16E-12	1.41E-09	-1.28	33.15
Negative regulation of viral genome replication	GO:0045071	14/51	2.32E-10	4.22E-08	-1.47	32.52
T cell migration	GO:0072678	7/17	3.50E-07	2.58E-05	-2.16	32.18
Response to interferon-alpha	GO:0035455	8/18	2.36E-08	2.57E-06	-1.82	31.92
Regulation of interferon-gamma production	GO:0032649	11/44	5.87E-08	5.92E-06	-1.91	31.72
Antigen receptor-mediated signaling pathway	GO:0050851	35/258	1.25E-13	4.25E-11	-1.07	31.65
Positive regulation of T cell activation	GO:0050870	17/69	1.83E-11	4.15E-09	-1.25	30.81
T cell chemotaxis	GO:0010818	5/11	1.02E-05	3.98E-04	-2.68	30.79
Myeloid cell activation involved in immune response	GO:0002275	5/12	1.71E-05	6.05E-04	-2.71	29.79
Immunological synapse formation	GO:0001771	4/7	2.77E-05	8.56E-04	-2.82	29.55
Regulation of granulocyte differentiation	GO:0030852	5/11	1.02E-05	3.98E-04	-2.54	29.15
Negative regulation of peptidyl-tyrosine phosphorylation	GO:0050732	8/28	1.28E-06	7.93E-05	-2.13	28.85
Cellular response to cytokine stimulus	GO:0071345	44/457	1.66E-11	4.12E-09	-1.16	28.80
Regulation of apoptotic process	GO:0042981	54/816	7.67E-08	6.97E-06	-1.74	28.58
Antigen processing and presentation of peptide antigen via MHC class I	GO:0002474	9/29	1.23E-07	1.05E-05	-1.72	27.41
Negative regulation of viral life cycle	GO:1903901	14/62	3.84E-09	5.51E-07	-1.41	27.38
Antigen processing and presentation of exogenous peptide antigen via MHC class I, TAP-independent	GO:0002480	5/10	5.72E-06	2.94E-04	-2.27	27.36
Positive regulation of T cell proliferation	GO:0042102	14/62	3.84E-09	5.51E-07	-1.40	27.05
T cell activation	GO:0042110	17/89	1.32E-09	2.25E-07	-1.32	27.02
Positive regulation of tumor necrosis factor production	GO:0032760	11/48	1.55E-07	1.28E-05	-1.71	26.80
Negative regulation of dendritic cell apoptotic process	GO:2000669	4/8	5.40E-05	1.42E-03	-2.60	25.50
Positive regulation of neutrophil chemotaxis	GO:0090023	7/25	6.99E-06	3.32E-04	-2.14	25.46
Antigen processing and presentation of exogenous peptide antigen via MHC class I	GO:0042590	16/79	1.64E-09	2.62E-07	-1.26	25.41

Positive regulation of lymphocyte differentiation	GO:0045621	6/17	7.23E-06	3.34E-04	-2.15	25.39
Positive regulation of intracellular signal transduction	GO:1902533	38/480	8.69E-08	7.64E-06	-1.56	25.39
Regulation of viral genome replication	GO:0045069	14/64	5.97E-09	7.75E-07	-1.34	25.39
Positive regulation of interferon-gamma production	GO:0032729	10/44	6.21E-07	4.23E-05	-1.77	25.36
Positive regulation of interleukin-6 secretion	GO:2000778	6/18	1.06E-05	4.05E-04	-2.21	25.28
Response to lipopolysaccharide	GO:0032496	19/156	2.94E-07	2.22E-05	-1.67	25.11
Positive regulation of apoptotic process	GO:0043065	28/308	2.84E-07	2.21E-05	-1.65	24.83
Regulation of dendritic cell apoptotic process	GO:2000668	5/11	1.02E-05	3.98E-04	-2.15	24.76
Regulation of T cell activation	GO:0050863	11/45	7.55E-08	6.97E-06	-1.51	24.74
Transmembrane receptor protein tyrosine kinase signaling pathway	GO:0007169	32/397	6.19E-07	4.23E-05	-1.69	24.10
Regulation of tumor necrosis factor secretion	GO:1904467	5/16	8.51E-05	2.02E-03	-2.57	24.04
B cell mediated immunity	GO:0019724	4/9	9.49E-05	2.17E-03	-2.58	23.93
Negative regulation of lymphocyte activation	GO:0051250	6/20	2.09E-05	6.96E-04	-2.22	23.88
Regulation of interleukin-6 production	GO:0032675	9/43	4.74E-06	2.53E-04	-1.91	23.46
Regulation of tumor necrosis factor production	GO:0032680	13/62	3.60E-08	3.77E-06	-1.37	23.40
Response to interferon-gamma	GO:0034341	14/69	1.68E-08	2.08E-06	-1.29	23.06
Proteolysis involved in cellular protein catabolic process	GO:0051603	12/94	2.79E-05	8.56E-04	-2.16	22.67
Positive regulation of B cell proliferation	GO:0030890	6/31	2.99E-04	5.36E-03	-2.77	22.49
Apoptotic process	GO:0006915	21/232	9.24E-06	3.89E-04	-1.94	22.47
Antigen processing and presentation of exogenous peptide antigen via MHC class II	GO:0019886	13/98	8.53E-06	3.69E-04	-1.90	22.14
Positive regulation of neutrophil migration	GO:1902624	7/26	9.31E-06	3.89E-04	-1.91	22.10
Positive regulation of granulocyte chemotaxis	GO:0071624	7/27	1.22E-05	4.64E-04	-1.92	21.72
Peptidyl-tyrosine autophosphorylation	GO:0038083	9/46	8.54E-06	3.69E-04	-1.86	21.70
Cellular response to lipopolysaccharide	GO:0071222	13/92	4.19E-06	2.28E-04	-1.74	21.52
Positive regulation of tumor necrosis factor secretion	GO:1904469	4/12	3.47E-04	6.18E-03	-2.70	21.51
Neutrophil degranulation	GO:0043312	33/480	1.28E-05	4.79E-04	-1.90	21.38
Antigen processing and presentation of exogenous peptide antigen	GO:0002478	13/98	8.53E-06	3.69E-04	-1.83	21.36
Negative regulation of leukocyte apoptotic process	GO:2000107	5/13	2.71E-05	8.56E-04	-2.02	21.21
Enzyme linked receptor protein signaling pathway	GO:0007167	15/121	4.15E-06	2.28E-04	-1.70	21.13
Positive regulation of interleukin-8 secretion	GO:2000484	5/15	6.00E-05	1.51E-03	-2.16	20.98
Antigen processing and presentation of exogenous peptide antigen via MHC class I, TAP-dependent	GO:0002479	14/76	6.13E-08	5.97E-06	-1.24	20.58
Antigen processing and presentation of peptide antigen via MHC class II	GO:0002495	13/99	9.55E-06	3.89E-04	-1.78	20.57
Positive regulation of response to cytokine stimulus	GO:0060760	5/15	6.00E-05	1.51E-03	-2.10	20.44
Regulation of intracellular signal transduction	GO:1902531	30/423	1.76E-05	6.14E-04	-1.85	20.24
Dendritic cell chemotaxis	GO:0002407	5/17	1.18E-04	2.58E-03	-2.23	20.15
Neutrophil mediated immunity	GO:0002446	33/488	1.79E-05	6.19E-04	-1.82	19.93
Antigen processing and presentation of endogenous peptide antigen	GO:0002483	3/7	8.97E-04	1.20E-02	-2.83	19.83
Regulation of cell proliferation	GO:0042127	52/741	2.06E-08	2.34E-06	-1.11	19.70

Negative regulation of cytokine production	GO:0001818	16/111	2.51E-07	2.01E-05	-1.30	19.70
Alpha-beta T cell activation	GO:0046631	3/7	8.97E-04	1.20E-02	-2.80	19.68
Cellular response to exogenous dsRNA	GO:0071360	4/14	6.68E-04	9.89E-03	-2.69	19.63
Peptidyl-tyrosine phosphorylation	GO:0018108	13/80	8.26E-07	5.36E-05	-1.40	19.55
Positive regulation of B cell differentiation	GO:0045579	3/8	1.40E-03	1.67E-02	-2.91	19.11
Positive regulation of programmed cell death	GO:0043068	25/258	3.74E-07	2.68E-05	-1.27	18.72
Positive regulation of cytokine-mediated signaling pathway	GO:0001961	7/34	6.16E-05	1.54E-03	-1.91	18.54
Positive regulation of interleukin-8 production	GO:0032757	9/45	7.06E-06	3.32E-04	-1.56	18.51
Response to type I interferon	GO:0034340	3/8	1.40E-03	1.67E-02	-2.81	18.47
Regulation of endothelial cell proliferation	GO:0001936	13/80	8.26E-07	5.36E-05	-1.32	18.45
Positive regulation of protein phosphorylation	GO:0001934	29/413	2.91E-05	8.80E-04	-1.76	18.34
Negative regulation of endothelial cell proliferation	GO:0001937	6/31	2.99E-04	5.36E-03	-2.25	18.26
Regulation of neutrophil chemotaxis	GO:0090022	7/30	2.59E-05	8.32E-04	-1.73	18.25
Regulation of nuclease activity	GO:0032069	3/7	8.97E-04	1.20E-02	-2.57	18.04
Positive regulation of kinase activity	GO:0033674	13/114	4.41E-05	1.24E-03	-1.80	18.01
Positive regulation of smooth muscle cell migration	GO:0014911	3/11	3.86E-03	3.42E-02	-3.24	18.01
Fusion of virus membrane with host plasma membrane	GO:0019064	3/8	1.40E-03	1.67E-02	-2.74	18.00
Regulation of B cell proliferation	GO:0030888	8/44	4.75E-05	1.31E-03	-1.80	17.94
Positive regulation of ERK1 and ERK2 cascade	GO:0070374	19/202	1.42E-05	5.24E-04	-1.61	17.92
Cellular defense response	GO:0006968	9/57	5.18E-05	1.37E-03	-1.81	17.83
Myeloid leukocyte differentiation	GO:0002573	10/51	2.64E-06	1.53E-04	-1.38	17.79
Non-canonical Wnt signaling pathway	GO:0035567	14/128	3.63E-05	1.05E-03	-1.73	17.68
Regulation of lymphocyte apoptotic process	GO:0070228	3/7	8.97E-04	1.20E-02	-2.51	17.64
Positive regulation of binding	GO:0051099	13/82	1.11E-06	7.01E-05	-1.28	17.62
Negative regulation of T cell activation	GO:0050868	7/35	7.50E-05	1.83E-03	-1.84	17.47
Regulation of interleukin-8 secretion	GO:2000482	6/24	6.55E-05	1.62E-03	-1.80	17.30
Regulation of small GTPase mediated signal transduction	GO:0051056	17/141	1.42E-06	8.60E-05	-1.28	17.27
Macropinocytosis	GO:0044351	3/7	8.97E-04	1.20E-02	-2.42	17.01
Leukocyte cell-cell adhesion	GO:0007159	6/28	1.65E-04	3.39E-03	-1.93	16.80
Lymphocyte chemotaxis	GO:0048247	9/45	7.06E-06	3.32E-04	-1.41	16.78
Cellular response to interleukin-15	GO:0071350	4/14	6.68E-04	9.89E-03	-2.29	16.77
Positive regulation of phosphorylation	GO:0042327	18/209	7.60E-05	1.83E-03	-1.76	16.66
Neutrophil chemotaxis	GO:0030593	9/54	3.32E-05	9.83E-04	-1.60	16.50
Positive regulation of tumor necrosis factor superfamily cytokine production	GO:1903557	7/33	5.03E-05	1.37E-03	-1.66	16.41
Positive regulation of interferon-alpha production	GO:0032727	5/20	2.73E-04	5.03E-03	-1.99	16.31
Membrane fusion involved in viral entry into host cell	GO:0039663	3/8	1.40E-03	1.67E-02	-2.48	16.26
Response to interferon-beta	GO:0035456	5/20	2.73E-04	5.03E-03	-1.98	16.22
Regulation of viral entry into host cell	GO:0046596	6/27	1.33E-04	2.86E-03	-1.82	16.21
Positive regulation of leukocyte chemotaxis	GO:0002690	10/62	1.64E-05	5.89E-04	-1.47	16.16
Collagen fibril organization	GO:0030199	7/30	2.59E-05	8.32E-04	-1.52	16.05
Positive regulation of defense response to virus by host	GO:0002230	5/22	4.41E-04	7.35E-03	-2.07	15.97
Cellular response to interferon-alpha	GO:0035457	3/8	1.40E-03	1.67E-02	-2.43	15.96
Peripheral nervous system axon ensheathment	GO:0032292	3/9	2.06E-03	2.18E-02	-2.56	15.85
T cell differentiation	GO:0030217	7/39	1.54E-04	3.26E-03	-1.81	15.85
Sensory organ morphogenesis	GO:0090596	3/8	1.40E-03	1.67E-02	-2.38	15.66
Positive regulation of cytokine secretion	GO:0050715	12/74	2.23E-06	1.32E-04	-1.20	15.62

Regulation of innate immune response	GO:0045088	11/70	8.08E-06	3.67E-04	-1.33	15.57
Mature B cell differentiation involved in immune response	GO:0002313	3/10	2.87E-03	2.78E-02	-2.65	15.51
Positive regulation of alpha-beta T cell proliferation	GO:0046641	4/14	6.68E-04	9.89E-03	-2.11	15.45
Positive regulation of cAMP-mediated signaling	GO:0043950	3/9	2.06E-03	2.18E-02	-2.49	15.42
Cell-matrix adhesion	GO:0007160	12/91	2.01E-05	6.83E-04	-1.42	15.41
Protein autophosphorylation	GO:0046777	16/176	9.98E-05	2.27E-03	-1.67	15.39
Regulation of defense response to virus by virus	GO:0050690	7/29	2.04E-05	6.87E-04	-1.42	15.34
Negative regulation of viral process	GO:0048525	5/22	4.41E-04	7.35E-03	-1.97	15.26
Adaptive immune response based on somatic recombination of immune receptors built from immunoglobulin superfamily domains	GO:0002460	4/15	8.89E-04	1.20E-02	-2.17	15.26
Fc-gamma receptor signaling pathway	GO:0038094	13/135	2.49E-04	4.79E-03	-1.84	15.24
Regulation of phosphatidylinositol 3-kinase signaling	GO:0014066	12/81	5.93E-06	2.98E-04	-1.26	15.22
Regulation of smooth muscle cell proliferation	GO:0048660	8/45	5.62E-05	1.45E-03	-1.54	15.11
Peptidyl-tyrosine modification	GO:0018212	8/52	1.63E-04	3.39E-03	-1.71	14.90
Regulation of cytokine production	GO:0001817	14/109	5.68E-06	2.94E-04	-1.23	14.87
Negative regulation of tumor necrosis factor production	GO:0032720	6/36	6.95E-04	1.02E-02	-2.03	14.79
Positive regulation of mononuclear cell migration	GO:0071677	5/20	2.73E-04	5.03E-03	-1.80	14.78
Branching involved in blood vessel morphogenesis	GO:0001569	4/15	8.89E-04	1.20E-02	-2.10	14.77
Positive regulation of immune response	GO:0050778	9/55	3.86E-05	1.11E-03	-1.45	14.74
Positive regulation of interleukin-6 production	GO:0032755	8/44	4.75E-05	1.31E-03	-1.47	14.67
Regulation of angiogenesis	GO:0045765	16/178	1.14E-04	2.53E-03	-1.61	14.66
Regulation of chemokine secretion	GO:0090196	3/9	2.06E-03	2.18E-02	-2.36	14.58
Regulated exocytosis	GO:0045055	13/149	6.48E-04	9.81E-03	-1.98	14.57
Schwann cell development	GO:0014044	3/9	2.06E-03	2.18E-02	-2.35	14.52
Neutrophil activation involved in immune response	GO:0002283	34/484	6.00E-06	2.98E-04	-1.21	14.49
Negative regulation of Wnt signaling pathway	GO:0030178	16/175	9.33E-05	2.16E-03	-1.56	14.48
Regulation of monocyte chemotaxis	GO:0090025	5/23	5.49E-04	8.71E-03	-1.92	14.38
Response to molecule of bacterial origin	GO:0002237	13/99	9.55E-06	3.89E-04	-1.24	14.37
T-helper 1 type immune response	GO:0042088	3/8	1.40E-03	1.67E-02	-2.18	14.29
Positive regulation of calcium ion transmembrane transport	GO:1904427	5/26	9.96E-04	1.31E-02	-2.06	14.24
Cell morphogenesis	GO:0000902	8/60	4.47E-04	7.35E-03	-1.84	14.22
Positive regulation of cell adhesion mediated by integrin	GO:0033630	4/15	8.89E-04	1.20E-02	-2.01	14.09
Negative regulation of type I interferon production	GO:0032480	8/45	5.62E-05	1.45E-03	-1.42	13.94
Positive regulation of defense response	GO:0031349	11/75	1.59E-05	5.79E-04	-1.26	13.93
Regulation of defense response to virus by host	GO:0050691	6/31	2.99E-04	5.36E-03	-1.71	13.91
Positive regulation of epithelial cell differentiation	GO:0030858	4/20	2.80E-03	2.74E-02	-2.34	13.77
Negative regulation of lymphocyte proliferation	GO:0050672	6/30	2.47E-04	4.78E-03	-1.65	13.72
Positive regulation of epidermis development	GO:0045684	3/10	2.87E-03	2.78E-02	-2.34	13.70
Positive regulation of myeloid leukocyte differentiation	GO:0002763	6/33	4.27E-04	7.32E-03	-1.76	13.66

Regulation of cysteine-type endopeptidase activity	GO:2000116	6/37	8.09E-04	1.17E-02	-1.92	13.64
Positive regulation of leukocyte cell-cell adhesion	GO:1903039	5/22	4.41E-04	7.35E-03	-1.76	13.59
Calcium ion regulated exocytosis	GO:0017156	3/12	5.03E-03	4.14E-02	-2.55	13.50
Pinocytosis	GO:0006907	3/10	2.87E-03	2.78E-02	-2.30	13.45
Positive regulation of protein binding	GO:0032092	9/61	8.95E-05	2.09E-03	-1.40	13.09
Tumor necrosis factor-mediated signaling pathway	GO:0033209	14/124	2.54E-05	8.32E-04	-1.23	12.99
Positive regulation of T cell apoptotic process	GO:0070234	3/11	3.86E-03	3.42E-02	-2.33	12.97
Negative regulation of canonical Wnt signaling pathway	GO:0090090	15/149	5.13E-05	1.37E-03	-1.31	12.94
Regulation of interferon-alpha production	GO:0032647	4/17	1.48E-03	1.72E-02	-1.98	12.91
Cellular protein modification process	GO:0006464	51/1002	2.37E-04	4.63E-03	-1.55	12.91
Positive regulation of cell proliferation	GO:0008284	28/425	1.19E-04	2.60E-03	-1.40	12.68
Immune response-activating cell surface receptor signaling pathway	GO:0002429	4/21	3.37E-03	3.12E-02	-2.23	12.67
Positive regulation of interleukin-4 production	GO:0032753	4/16	1.16E-03	1.47E-02	-1.86	12.58
Positive regulation of cyclic nucleotide metabolic process	GO:0030801	4/17	1.48E-03	1.72E-02	-1.93	12.58
Cellular response to tumor necrosis factor	GO:0071356	18/195	3.06E-05	9.16E-04	-1.21	12.56
Positive regulation of interleukin-1 beta production	GO:0032731	6/32	3.59E-04	6.32E-03	-1.58	12.54
Dendritic cell differentiation	GO:0097028	5/22	4.41E-04	7.35E-03	-1.62	12.50
Positive regulation of focal adhesion assembly	GO:0051894	4/18	1.85E-03	2.03E-02	-1.98	12.47
Granulocyte chemotaxis	GO:0071621	9/57	5.18E-05	1.37E-03	-1.26	12.47
Negative regulation of peptide secretion	GO:0002792	3/12	5.03E-03	4.14E-02	-2.35	12.43
Positive regulation of lymphocyte migration	GO:2000403	4/18	1.85E-03	2.03E-02	-1.97	12.39
Cellular response to dsRNA	GO:0071359	4/17	1.48E-03	1.72E-02	-1.90	12.38
Positive regulation of phosphatidylinositol 3-kinase signaling	GO:0014068	8/54	2.13E-04	4.26E-03	-1.46	12.31
Regulation of blood coagulation	GO:0030193	5/31	2.27E-03	2.36E-02	-2.01	12.26
Regulation of transcription factor import into nucleus	GO:0042990	4/20	2.80E-03	2.74E-02	-2.08	12.21
Heterotypic cell-cell adhesion	GO:0034113	5/26	9.96E-04	1.31E-02	-1.77	12.20
Positive regulation of interleukin-1 beta secretion	GO:0050718	5/26	9.96E-04	1.31E-02	-1.74	12.01
Regulation of dendritic cell chemotaxis	GO:2000508	3/7	8.97E-04	1.20E-02	-1.71	11.99
Monocyte differentiation	GO:0030224	3/12	5.03E-03	4.14E-02	-2.26	11.95
Positive regulation of dendritic cell chemotaxis	GO:2000510	3/8	1.40E-03	1.67E-02	-1.82	11.95
Cell projection assembly	GO:0030031	4/21	3.37E-03	3.12E-02	-2.10	11.95
Response to lipid	GO:0033993	14/141	1.05E-04	2.37E-03	-1.29	11.86
Positive regulation of monocyte chemotaxis	GO:0090026	4/18	1.85E-03	2.03E-02	-1.88	11.81
Negative regulation of cytokine secretion	GO:0050710	7/41	2.14E-04	4.26E-03	-1.40	11.81
Positive regulation of protein kinase B signaling	GO:0051897	14/128	3.63E-05	1.05E-03	-1.15	11.72
Negative regulation of morphogenesis of an epithelium	GO:1905331	3/12	5.03E-03	4.14E-02	-2.21	11.70
Positive regulation of transcription factor import into nucleus	GO:0042993	6/33	4.27E-04	7.32E-03	-1.51	11.70
Regulation of podosome assembly	GO:0071801	3/11	3.86E-03	3.42E-02	-2.10	11.67
Regulation of smooth muscle cell migration	GO:0014910	4/19	2.29E-03	2.37E-02	-1.91	11.61
Positive regulation of homeostatic process	GO:0032846	9/84	1.02E-03	1.33E-02	-1.68	11.59
Macrophage differentiation	GO:0030225	3/12	5.03E-03	4.14E-02	-2.18	11.54
JAK-STAT cascade	GO:0007259	6/41	1.41E-03	1.67E-02	-1.75	11.50
Regulation of interleukin-4 production	GO:0032673	4/16	1.16E-03	1.47E-02	-1.70	11.50
Neutrophil migration	GO:1990266	9/59	6.85E-05	1.68E-03	-1.19	11.42

Myeloid dendritic cell activation	GO:0001773	4/15	8.89E-04	1.20E-02	-1.62	11.41
Positive regulation of leukocyte migration	GO:0002687	6/35	5.94E-04	9.10E-03	-1.53	11.33
Regulation of Ras protein signal transduction	GO:0046578	9/91	1.81E-03	2.03E-02	-1.79	11.28
Interleukin-15-mediated signaling pathway	GO:0035723	4/14	6.68E-04	9.89E-03	-1.54	11.26
Regulation of signal transduction	GO:0009966	19/234	1.07E-04	2.38E-03	-1.23	11.26
Regulation of transmembrane receptor protein serine/threonine kinase signaling pathway	GO:0090092	5/29	1.67E-03	1.89E-02	-1.76	11.23
Regulation of peptidyl-tyrosine phosphorylation	GO:0050730	9/86	1.21E-03	1.53E-02	-1.67	11.21
Positive regulation of actin filament polymerization	GO:0030838	8/52	1.63E-04	3.39E-03	-1.28	11.19
Negative regulation of cell proliferation	GO:0008285	23/364	8.35E-04	1.19E-02	-1.57	11.11
B cell activation	GO:0042113	9/74	4.02E-04	7.03E-03	-1.41	11.05
Negative regulation of angiogenesis	GO:0016525	9/70	2.63E-04	4.99E-03	-1.33	10.98
Positive regulation of phospholipase activity	GO:0010518	4/20	2.80E-03	2.74E-02	-1.86	10.93
Vascular endothelial growth factor receptor signaling pathway	GO:0048010	9/71	2.94E-04	5.36E-03	-1.34	10.88
Negative regulation of leukocyte cell-cell adhesion	GO:1903038	3/12	5.03E-03	4.14E-02	-2.05	10.86
Positive regulation of interferon-gamma biosynthetic process	GO:0045078	3/12	5.03E-03	4.14E-02	-2.04	10.81
Regulation of protein tyrosine kinase activity	GO:0061097	5/32	2.62E-03	2.62E-02	-1.82	10.80
Positive regulation of viral life cycle	GO:1903902	6/45	2.31E-03	2.37E-02	-1.78	10.79
Negative regulation of immune response	GO:0050777	6/45	2.31E-03	2.37E-02	-1.77	10.76
Positive regulation of transcription, DNA-templated	GO:0045893	54/1121	5.89E-04	9.10E-03	-1.43	10.66
Regulation of DNA binding	GO:0051101	8/53	1.87E-04	3.77E-03	-1.24	10.61
Regulation of response to cytokine stimulus	GO:0060759	4/21	3.37E-03	3.12E-02	-1.86	10.61
Regulation of T cell apoptotic process	GO:0070232	3/11	3.86E-03	3.42E-02	-1.89	10.51
Cellular response to molecule of bacterial origin	GO:0071219	10/85	2.56E-04	4.87E-03	-1.27	10.49
Positive regulation of T cell migration	GO:2000406	4/21	3.37E-03	3.12E-02	-1.84	10.47
Substrate adhesion-dependent cell spreading	GO:0034446	5/33	3.02E-03	2.86E-02	-1.80	10.45
Protein complex subunit organization	GO:0071822	7/46	4.47E-04	7.35E-03	-1.36	10.45
Regulation of epidermal cell differentiation	GO:0045604	4/21	3.37E-03	3.12E-02	-1.83	10.41
Positive regulation of viral entry into host cell	GO:0046598	3/9	2.06E-03	2.18E-02	-1.66	10.24
Regulation of erythrocyte differentiation	GO:0045646	6/33	4.27E-04	7.32E-03	-1.31	10.13
Mononuclear cell differentiation	GO:1903131	3/12	5.03E-03	4.14E-02	-1.91	10.11
Positive regulation of interleukin-1 secretion	GO:0050716	5/30	1.95E-03	2.13E-02	-1.62	10.08
Positive regulation of purine nucleotide metabolic process	GO:1900544	4/15	8.89E-04	1.20E-02	-1.43	10.07
Negative regulation of apoptotic process	GO:0043066	32/486	4.03E-05	1.14E-03	-0.99	10.04
Positive regulation of adherens junction organization	GO:1903393	4/20	2.80E-03	2.74E-02	-1.70	10.00
Negative regulation of blood vessel morphogenesis	GO:2000181	9/66	1.67E-04	3.40E-03	-1.15	9.99
Hemopoiesis	GO:0030097	9/77	5.41E-04	8.68E-03	-1.32	9.94
Positive regulation of nucleic acid-templated transcription	GO:1903508	28/503	1.69E-03	1.91E-02	-1.56	9.93
Cell morphogenesis involved in differentiation	GO:0000904	7/55	1.34E-03	1.66E-02	-1.49	9.87
Skeletal system development	GO:0001501	13/147	5.70E-04	8.92E-03	-1.32	9.84
Regulation of G1/S transition of mitotic cell cycle	GO:2000045	6/47	2.89E-03	2.78E-02	-1.68	9.84

Cellular response to lipid	GO:0071396	16/179	1.22E-04	2.63E-03	-1.09	9.78
Regulation of leukocyte cell-cell adhesion	GO:1903037	3/12	5.03E-03	4.14E-02	-1.85	9.77
Regulation of interleukin-1 beta secretion	GO:0050706	6/35	5.94E-04	9.10E-03	-1.31	9.74
Positive regulation of peptidyl-tyrosine phosphorylation	GO:0050731	12/117	2.38E-04	4.63E-03	-1.17	9.73
Positive regulation of inflammatory response	GO:0050729	8/74	1.82E-03	2.03E-02	-1.54	9.70
Interleukin-35-mediated signaling pathway	GO:0070757	3/12	5.03E-03	4.14E-02	-1.83	9.69
Positive regulation of cytoskeleton organization	GO:0051495	8/78	2.54E-03	2.57E-02	-1.62	9.69
Positive regulation of release of sequestered calcium ion into cytosol	GO:0051281	5/32	2.62E-03	2.62E-02	-1.63	9.67
Positive regulation of MAPK cascade	GO:0043410	22/290	8.63E-05	2.03E-03	-1.03	9.63
Response to exogenous dsRNA	GO:0043330	5/34	3.45E-03	3.12E-02	-1.69	9.58
Regulation of GTPase activity	GO:0043087	16/189	2.28E-04	4.51E-03	-1.13	9.50
Regulation of T cell migration	GO:2000404	4/18	1.85E-03	2.03E-02	-1.50	9.44
Negative regulation of macromolecule metabolic process	GO:0010605	11/125	1.54E-03	1.76E-02	-1.46	9.44
Negative regulation of antigen receptor-mediated signaling pathway	GO:0050858	4/18	1.85E-03	2.03E-02	-1.50	9.42
Positive regulation of chemokine production	GO:0032722	6/35	5.94E-04	9.10E-03	-1.27	9.40
Plasma membrane organization	GO:0007009	5/37	5.01E-03	4.14E-02	-1.77	9.37
Regulation of protein phosphorylation	GO:0001932	20/262	1.64E-04	3.39E-03	-1.07	9.34
Regulation of ERK1 and ERK2 cascade	GO:0070372	20/248	7.78E-05	1.86E-03	-0.99	9.34
Positive regulation of response to external stimulus	GO:0032103	10/91	4.46E-04	7.35E-03	-1.21	9.32
STAT cascade	GO:0097696	4/23	4.75E-03	4.08E-02	-1.74	9.31
Negative regulation of epithelial cell proliferation	GO:0050680	8/67	9.47E-04	1.26E-02	-1.33	9.23
Positive regulation of cellular component movement	GO:0051272	4/22	4.02E-03	3.48E-02	-1.65	9.12
Fc receptor signaling pathway	GO:0038093	15/184	5.29E-04	8.53E-03	-1.20	9.06
Positive regulation of erythrocyte differentiation	GO:0045648	4/23	4.75E-03	4.08E-02	-1.69	9.05
Activation of MAPKK activity	GO:0000186	5/34	3.45E-03	3.12E-02	-1.60	9.05
Positive regulation of phospholipase C activity	GO:0010863	5/31	2.27E-03	2.36E-02	-1.48	9.04
Negative regulation of response to stimulus	GO:0048585	12/128	5.45E-04	8.68E-03	-1.20	9.02
Negative regulation of programmed cell death	GO:0043069	26/409	3.59E-04	6.32E-03	-1.14	9.02
Positive regulation of chemokine secretion	GO:0090197	3/9	2.06E-03	2.18E-02	-1.45	8.95
Positive regulation of cell junction assembly	GO:1901890	4/24	5.57E-03	4.53E-02	-1.72	8.94
Fc receptor mediated stimulatory signaling pathway	GO:0002431	13/136	2.68E-04	5.03E-03	-1.08	8.91
Bone development	GO:0060348	5/35	3.92E-03	3.44E-02	-1.61	8.90
Positive regulation of endothelial cell proliferation	GO:0001938	8/68	1.05E-03	1.35E-02	-1.29	8.88
Positive regulation of gene expression	GO:0010628	39/772	1.45E-03	1.71E-02	-1.34	8.79
Regulation of membrane protein ectodomain proteolysis	GO:0051043	4/23	4.75E-03	4.08E-02	-1.64	8.76
Positive regulation of calcium ion transport into cytosol	GO:0010524	5/33	3.02E-03	2.86E-02	-1.50	8.72
Positive regulation of transcription from RNA polymerase II promoter	GO:0045944	41/849	2.53E-03	2.56E-02	-1.45	8.70
Positive regulation of protein modification process	GO:0031401	15/164	1.52E-04	3.25E-03	-0.99	8.69
Negative regulation of tumor necrosis factor superfamily cytokine production	GO:1903556	5/32	2.62E-03	2.62E-02	-1.46	8.67
B cell homeostasis	GO:0001782	3/10	2.87E-03	2.78E-02	-1.48	8.65
Regulation of protein kinase B signaling	GO:0051896	14/164	5.07E-04	8.23E-03	-1.14	8.63

Regulation of I-kappaB kinase/NF-kappaB signaling	GO:0043122	16/205	5.65E-04	8.90E-03	-1.15	8.62
Regulation of actin filament polymerization	GO:0030833	8/69	1.15E-03	1.47E-02	-1.27	8.56
Negative regulation of signaling	GO:0023057	9/84	1.02E-03	1.33E-02	-1.23	8.45
Regulation of T cell differentiation	GO:0045580	5/34	3.45E-03	3.12E-02	-1.49	8.43
Positive regulation of cell-matrix adhesion	GO:0001954	5/34	3.45E-03	3.12E-02	-1.48	8.38
Fc-epsilon receptor signaling pathway	GO:0038095	15/183	4.99E-04	8.15E-03	-1.10	8.35
Positive regulation of endocytosis	GO:0045807	8/65	7.73E-04	1.13E-02	-1.16	8.34
Regulation of release of sequestered calcium ion into cytosol	GO:0051279	6/50	3.96E-03	3.44E-02	-1.50	8.28
Positive regulation of hydrolase activity	GO:0051345	13/173	2.52E-03	2.56E-02	-1.38	8.28
Positive regulation of cellular metabolic process	GO:0031325	9/93	2.10E-03	2.22E-02	-1.32	8.16
Regulation of canonical Wnt signaling pathway	GO:0060828	16/214	8.98E-04	1.20E-02	-1.16	8.15
Positive regulation of smooth muscle cell proliferation	GO:0048661	5/38	5.63E-03	4.57E-02	-1.57	8.13
Innate immune response activating cell surface receptor signaling pathway	GO:0002220	11/125	1.54E-03	1.76E-02	-1.24	8.06
Regulation of type I interferon production	GO:0032479	9/86	1.21E-03	1.53E-02	-1.20	8.05
Entry into host cell	GO:0030260	5/34	3.45E-03	3.12E-02	-1.41	7.98
Positive regulation of cytokine biosynthetic process	GO:0042108	6/41	1.41E-03	1.67E-02	-1.21	7.92
Integrin-mediated signaling pathway	GO:0007229	7/59	2.03E-03	2.18E-02	-1.28	7.92
Regulation of cell migration	GO:0030334	21/317	7.63E-04	1.12E-02	-1.10	7.90
Regulation of phosphatidylinositol 3-kinase activity	GO:0043551	5/34	3.45E-03	3.12E-02	-1.38	7.83
Endocytosis	GO:0006897	17/264	3.13E-03	2.97E-02	-1.35	7.81
Fc-gamma receptor signaling pathway involved in phagocytosis	GO:0038096	12/134	8.21E-04	1.17E-02	-1.10	7.79
Stimulatory C-type lectin receptor signaling pathway	GO:0002223	11/122	1.26E-03	1.58E-02	-1.16	7.76
Regulation of protein binding	GO:0043393	10/112	2.23E-03	2.34E-02	-1.26	7.67
Regulation of MAPK cascade	GO:0043408	15/204	1.52E-03	1.76E-02	-1.18	7.64
Negative regulation of sequence-specific DNA binding transcription factor activity	GO:0043433	11/141	3.96E-03	3.44E-02	-1.38	7.64
Negative regulation of DNA binding	GO:0043392	5/35	3.92E-03	3.44E-02	-1.37	7.58
Negative regulation of interferon-gamma production	GO:0032689	4/21	3.37E-03	3.12E-02	-1.32	7.51
Toll-like receptor signaling pathway	GO:0002224	9/87	1.32E-03	1.64E-02	-1.13	7.47
Wnt signaling pathway, calcium modulating pathway	GO:0007223	5/37	5.01E-03	4.14E-02	-1.40	7.43
Positive regulation of epithelial cell proliferation	GO:0050679	10/108	1.70E-03	1.91E-02	-1.16	7.42
Pattern recognition receptor signaling pathway	GO:0002221	6/49	3.58E-03	3.21E-02	-1.31	7.36
Cellular response to oxygen-containing compound	GO:1901701	19/275	8.13E-04	1.17E-02	-1.03	7.35
Negative regulation of cell migration	GO:0030336	11/122	1.26E-03	1.58E-02	-1.09	7.30
Positive regulation of JAK-STAT cascade	GO:0046427	7/65	3.54E-03	3.20E-02	-1.29	7.25
Cellular response to organic substance	GO:0071310	12/134	8.21E-04	1.17E-02	-1.02	7.22
Protein phosphorylation	GO:0006468	28/471	6.29E-04	9.59E-03	-0.97	7.14
Regulation of cysteine-type endopeptidase activity involved in apoptotic process	GO:0043281	9/94	2.26E-03	2.36E-02	-1.15	7.03
Positive regulation of tyrosine phosphorylation of STAT protein	GO:0042531	6/54	5.81E-03	4.67E-02	-1.36	7.01
Positive regulation of chemotaxis	GO:0050921	6/49	3.58E-03	3.21E-02	-1.24	6.99
Positive regulation of endothelial cell migration	GO:0010595	7/71	5.79E-03	4.67E-02	-1.31	6.75
Phosphorylation	GO:0016310	22/387	3.96E-03	3.44E-02	-1.21	6.68

Negative regulation of cysteine-type endopeptidase activity	GO:2000117	8/73	1.66E-03	1.89E-02	-1.01	6.49
Regulation of osteoblast differentiation	GO:0045667	7/70	5.35E-03	4.37E-02	-1.23	6.41
Positive regulation of cytosolic calcium ion concentration	GO:0007204	11/134	2.67E-03	2.66E-02	-1.08	6.41
Post-translational protein modification	GO:0043687	22/358	1.53E-03	1.76E-02	-0.99	6.39
Cellular protein catabolic process	GO:0044257	7/63	2.96E-03	2.84E-02	-1.09	6.36
Regulation of endopeptidase activity	GO:0052548	7/69	4.94E-03	4.14E-02	-1.19	6.31
Chemokine-mediated signaling pathway	GO:0070098	6/53	5.30E-03	4.34E-02	-1.19	6.23
Regulation of kinase activity	GO:0043549	9/102	3.94E-03	3.44E-02	-1.12	6.19
Negative regulation of cell motility	GO:2000146	9/98	3.01E-03	2.86E-02	-1.06	6.16
Positive regulation of GTPase activity	GO:0043547	14/193	2.43E-03	2.48E-02	-1.02	6.12
MAPK cascade	GO:0000165	18/279	2.36E-03	2.41E-02	-0.93	5.62
Vesicle organization	GO:0016050	10/128	5.81E-03	4.67E-02	-1.07	5.52
Regulation of cytosolic calcium ion concentration	GO:0051480	11/138	3.36E-03	3.12E-02	-0.96	5.46
Regulation of inflammatory response	GO:0050727	12/167	5.19E-03	4.26E-02	-0.81	4.24
Analysis based on significantly down-regulated genes						
GO term	GO ID	Overlap	P-value	Adjusted P-value	Z-score	Combined score
Protein targeting to ER	GO:0045047	11/98	1.95E-05	2.40E-02	-2.09	22.66
Nuclear-transcribed mRNA catabolic process, nonsense-mediated decay	GO:0000184	11/113	7.38E-05	2.40E-02	-2.00	19.00
SRP-dependent cotranslational protein targeting to membrane	GO:0006614	10/90	5.07E-05	2.40E-02	-1.84	18.19
Viral transcription	GO:0019083	11/114	7.99E-05	2.40E-02	-1.78	16.84
Viral gene expression	GO:0019080	11/111	6.26E-05	2.40E-02	-1.58	15.30
Cotranslational protein targeting to membrane	GO:0006613	10/94	7.37E-05	2.40E-02	-1.37	13.01
Cellular protein metabolic process	GO:0044267	27/485	3.61E-05	2.40E-02	-1.18	12.06
